# Digestive anatomy and diet of free-ranging maned wolf (*Chrysocyon brachyurus*)

**DOI:** 10.1007/s42991-025-00493-z

**Published:** 2025-04-24

**Authors:** Bruno Costa Silva, Luan Alexander de Oliveira, Marcus Clauss, Claudia Guimarães Costa, Leandro de Oliveira Marques Alexandre, María J. Duque-Correa

**Affiliations:** 1 Departamento de Medicina Veterinária – Pontifícia, Universidade Católica de Minas Gerais, Belo Horizonte, Brazil; 2 Programa de Pós-Graduação Em Biodiversidade E Meio Ambiente - Pontifícia Universidade Católica de Minas Gerais, Av. Dom José Gaspar, Belo Horizonte, 500 Brazil; 3 Clinic for Zoo Animals, Exotic Pets and Wildlife, Vetsuisse Faculty, University of Zurich, Winterthurerstrasse 260, 8057 Zurich, Switzerland; 4 Coleção de Mastozoologia, Museu de Ciências Naturais da Pontifícia Universidade Católica de Minas, Belo Horizonte, Brazil

**Keywords:** Anthropogenic material, Fiber, Gastrointestinal tract, Omnivore, Solanum lycocarpum

## Abstract

The maned wolf (*Chrysocyon brachyurus*) is the largest of the currently extant ten species of South American canids and is a monotypic taxon in the genus *Chrysocyon*, currently classified as Near Threatened by the IUCN. Here we report the anatomy of the gastrointestinal tract (GIT) of 12 free-ranging maned wolves (10 adults, six females, four males, and two juvenile males), nutritional analyses of the gastrointestinal tract (stomach, small, and large intestine) of eight individuals (six females and two males), and the macroscopical examination of the stomach contents of eight individuals (four adult females, two adult males, and two juvenile males). The general anatomy and the dimensions of the maned wolf`s GIT resembled that of other canids. The results of the dietary analyses reported in this study are similar to previously reported data for the species, confirming its omnivorous nature. Vertebrates were found in all stomachs, representing 42.5% of the total dietary mass. Vegetable material was also present in all stomachs, accounting for half of the total dietary mass (50.5%). Anthropogenic material, including cooked rice, glass, and ceramic fragments, were retrieved from the stomach of one individual; such opportunistic feeding behavior has been previously reported. When compared to other Carnivora species classified as omnivores, including the red fox (*Vulpes vulpes*) and the raccoon dog (*Nyctereutes procyonides*), these ‘omnivorous’ carnivores ingest distinctively higher levels of fiber compared to purely faunivorous Carnivora. This information might be important for feeding these species in *ex-situ* settings.

**Supplementary Information:**

The online version contains supplementary material available at 10.1007/s42991-025-00493-z.

## Introduction

The maned wolf (*Chrysocyon brachyurus*) is the largest of the currently extant ten species of South American canids, and is a monotypic taxon in the genus *Chrysocyon* (Dietz 1985; De Almeida Jácomo et al. 2004; Perini et al. 2010; Chavez et al. 2022). The historical distribution of the species included six countries: Argentina, Bolivia, Brazil, Paraguay, Perú, and Uruguay (Dietz 1985; Coelho et al. 2018). Currently, the majority (> 90%) of the population is estimated to be restricted to Brazil, and the species is possibly not present in Uruguay (Paula and DeMatteo 2015). The maned wolf´s risk of extinction is classified as Near Threatened by the International Union for Conservation of Nature (Paula and DeMatteo 2015; IUCN 2024).

Maned wolves are generalist omnivores; over 500 dietary items have been reported to be consumed by the species, with an average consumption of 50% vegetable material and 50% animal matter (Dietz 1985; Motta-Junior and Martins 2002; Santos et al. 2003; Silva and Talamoni 2003; Motta-Junior et al. 2013; reviewed in Weber et al. 2023). However, the relative importance of dietary items is seasonal and dependent on the habitat of the individuals (Dietz 1985; Aragona and Setz 2001; Motta-Junior et al. 2013). *Solanum lycocarpum*, commonly known as “Lobeira” or “wolf's fruit,” is the most commonly consumed plant material (Aragona and Setz 2001; Santos et al. 2003; Weber et al. 2023). Consumed animal matter includes a wide variety of species, such as small mammals (i.e., rodents and armadillos), birds, reptiles, and invertebrates (Dietz 1985; Motta‐Junior et al. 1996; Aragona and Setz 2001; Santos et al. 2003; De Almeida Jácomo et al. 2004; Motta-Junior et al. 2013).

The gastrointestinal tract of canids is comparable to that of other Carnivora; it is not very complex and relatively short, with a simple stomach, a small cecum, and an unsacculated colon (Dietz 1985; Motta‐Junior et al. 1996; Aragona and Setz 2001; Santos et al. 2003; De Almeida Jácomo et al. 2004; Motta-Junior et al. 2013). Few quantitative anatomical descriptions of the anatomy of the gastrointestinal tract of wild canids exist. McGrosky et al. (2016) report the intestinal length of several carnivoran species, including five canids. Further anatomical descriptions of canine digestive tract anatomy have been published for the raccoon dog (*Nyctereutes procyonoides*) (Kulawik et al. 2009), the Arctic fox (*Alopex lagopus*) (Brudnicki et al. 2011), and the grey fox (*Urocyon cinereoargenteus*) (Duque-Correa et al. 2021). The external morphology of the maned wolf has been described extensively (Dietz 1984; 1985); however, to date, descriptions of the gastrointestinal anatomy are lacking. Therefore, we report observations on the digestive anatomy of free-ranging maned wolves, including the macroscopic description of their stomach contents and the nutritional analyses of gastrointestinal contents.

## Materials and methods

### Specimen collection

A total of 15 animals, including nine adult females, four adult males, and two juvenile males, were collected between June 2021 and September 2022 (Table S1). All specimens were found dead, likely due to traffic collision, near or on main roads in the state of Minas Gerais, Brazil, and reported to the Coleção de Mastozoologia from the Museu de Ciencias Naturais of the Pontifícia Universidade Católica de Minas Gerais (MCN PUC Minas), where they were kept frozen (−20 ºC) until necropsy. Individuals were assigned an identification number, date, and geographic location (coordinates) where they were found. The sex was noted, and whether the individual was juvenile or adult (Table S1).

### Anatomical examination

Twelve animals underwent anatomical examination (females 1–6, males 1–4, and juvenile males 1 and 2, Table S1). The body mass was recorded, then the body cavity was accessed, and the whole digestive tract, from the esophagus to the anus, including the liver, was removed, photographed, and further examined. The liver was separated, and the mesenteries were removed from the gastrointestinal tract. We then measured the greater and lesser curvature of the stomach, the length of the small intestine, cecum, and colon/rectum (from here onwards, referred to as colon). The small intestine was measured from the stomach's pyloric sphincter to the ileocolic papillae (start of the cecum). The colon was measured from the ileocolic papillae (end of the cecum) to the anus. The total intestinal length corresponds to the added length of all intestinal sections, from the small intestine to the anus. Additionally, we measured the diameter of the GIT using a digital caliper (0.01 cm); stomach diameter was measured at the fundus, body, and pylorus, and the diameter of all intestinal sections is the average of three measurements per section taken at the start, middle, and end of each section. All organs were ligated at the cranial and caudal borders and separated. All organs were then weighed with contents using a digital scale. All organs were cut open, and the contents were collected for further analyses (see dietary analyses); finally, the organs were weighed empty. We calculated the average length of all organs for adult females and males, juveniles, and adults from both sexes. The average intestine length of the adult maned wolves was compared with published data (Kulawik et al. 2009; Brudnicki et al. 2011; McGrosky et al. 2016; Duque-Correa et al. 2021) for other carnivore species. We report descriptive statistics, the mean and standard deviation of all lengths and weights, and averages for adult females and males, juveniles, and adults.

### Dietary analyses

We analyzed the stomach contents of eight adults (females 1, 2, 4, 7–9, and males 1 and 4) (Table S1). The stomach was cut along the greater curvature. The contents were removed, packed in plastic bags and kept frozen until further analyses. After thawing, the contents were washed with running water in a sieve of 500 μm pore size, and then allowed to dry. All items were classified into one of six categories: vertebrates (hair, feathers, soft tissue, teeth, claws, beaks, and bones of birds and mammals), invertebrates (whole insects, arachnids), vegetable material (stems, leaves, seeds, and fruit pulp), anthropogenic content (edible or non-edible items related with human presence), mineral material, and non-identified material. The material per category was then weighed to calculate the relative weight composition (%W) as described by Hynes (1950), which is the proportion of biomass of a particular dietary category to the total biomass. We further calculated the frequency of occurrence (FO), that is, the proportion of non-empty stomachs containing a particular dietary category relative to all non-empty stomachs (Hynes 1950).

Nutritional analyses were done on representative samples for all individuals from which sufficient samples could be retrieved (females 1–2, 4–5, males 1–2, and juveniles 1–2); the contents were mixed to homogenize them, and an average of 15 g was saved for bromatological analyses. All small intestines contained at least 40 g, but given their high moisture content, final analyses were done on only five samples. Cecum content was not analyzed due to the low volumes present in the organ. One colon was empty, and material was insufficient in four others, so the final analyses were only done for six samples (Table S4). Dry matter (DM), crude protein (CP, Kjeldahl method, CP = 6.25 × nitrogen), neutral detergent fiber (NDF, without correcting for residual ash), ether extract (EE), and total ash were measured using standard methods (AOAC 2023), as routinely performed at the Animal Nutrition Laboratory of the Pontifícia Universidade Católica de Minas Gerais, Betim.

## Results

We examined the anatomy of 12 individuals: 10 adults (six females, and four males) and two juvenile males (Tables 1, 2, 3, S2, Figs. 1, S1–S12). Females had an average body mass of 20.8 ± 4.7 SD kg, males 19.2 ± 4.6 SD kg, and adults'combined average body mass was 20.1 ± 4.5 SD kg. The two juveniles had a body mass of 6.4 and 13.3 kg.

**Table 1:** Empty mass (in g; mean ± SD) of different components of the gastrointestinal tract (GIT) of maned wolves (*Chrysocyon brachyurus*)

	Juveniles^a^	Adults		
	(*n* = 2)	Females (*n* = 6)	Males (*n* = 4)	Average (*n* = 10)
Body mass (kg)	6.4/13.3	20.7 ± 4.7	19.2 ± 4.6	20.1 ± 4.5
Liver	130/347	329.3 ± 75.8	398.0 ± 49.9	356.8 ± 72.7
Stomach	65/150	187.2 ± 26.8	188.2 ± 47.3	187.6 ± 33.8
Small intestine	90/540	321.1 ± 82.7	445.0 ± 119.9	370.7 ± 112.6
Caecum	3/4	9.1 ± 5.4	12.5 ± 6.7	10.4 ± 5.9
Colon	25/100	124.6 ± 27.2	126.5 ± 37.7	125.4 ± 29.8
Large intestine	28/104	133.7 ± 28.8	139.0 ± 40.3	135.8 ± 31.8
Total intestine	118/644	454.9 ± 105.5	584.0 ± 153.2	506.5 ± 135.8
Total GIT	183/794	642.1 ± 124.8	772.2 ± 196.1	694.1 ± 161.2

^a^For the two juveniles, values of each animal are displayed

**Fig. 1:**
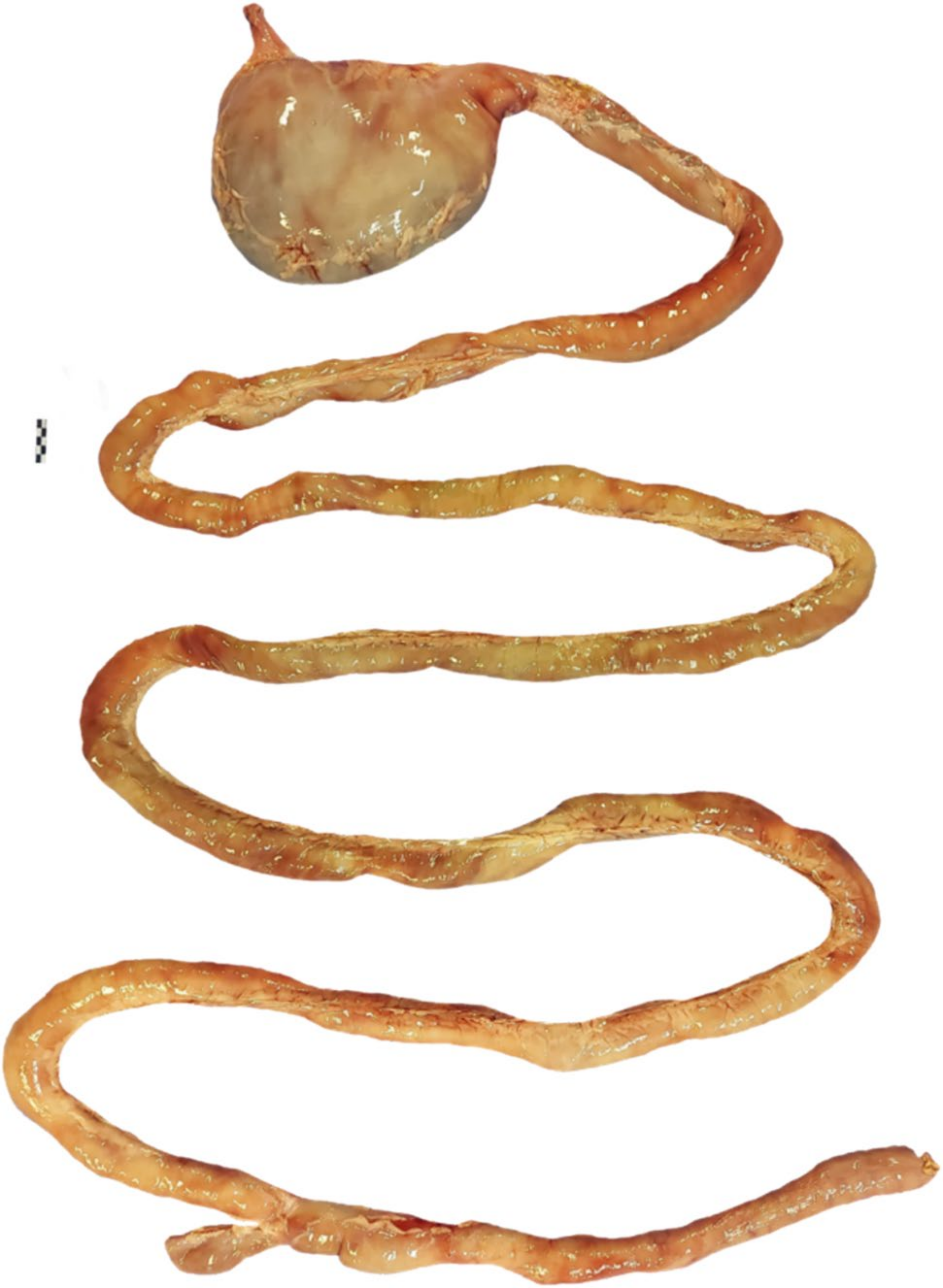
Gastrointestinal tract of a maned wolf (*Chrysocyon brachyurus*). Scale = 5 cm

**Fig. 2:**
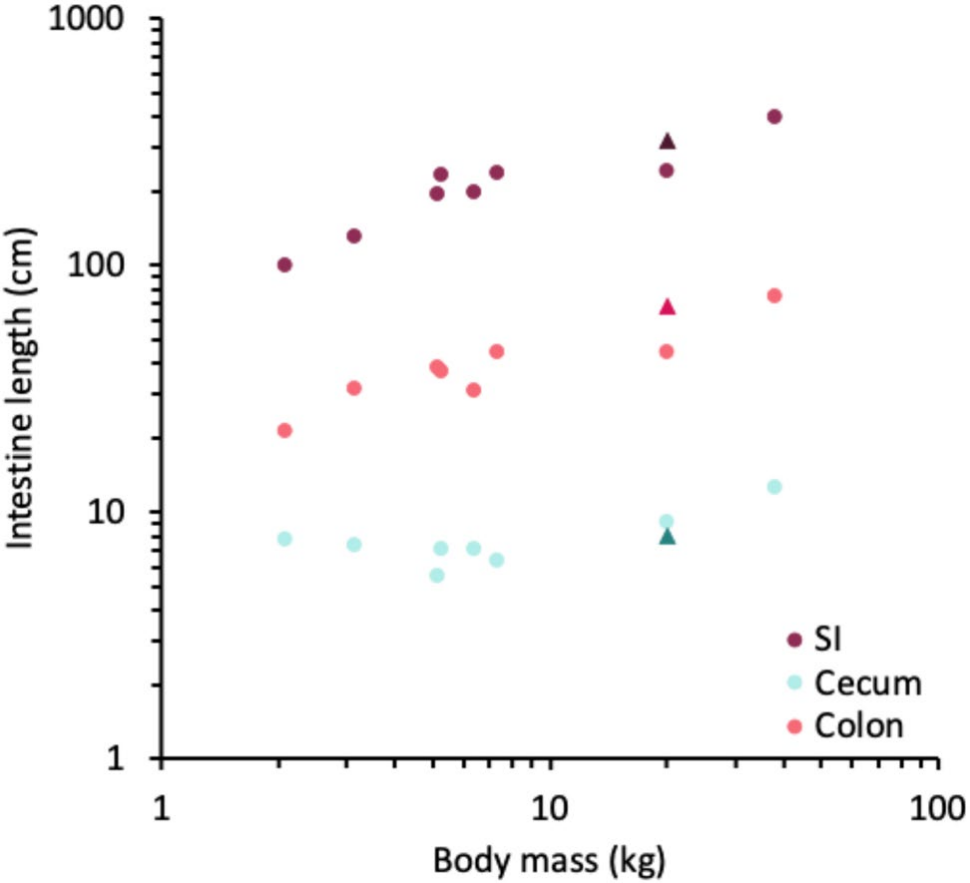
Length of the intestinal sections for various canid species (dots, in increasing body mass: *Vulpes corsac*, *Urocyon cinereoargeteus*, *Vulpes vulpes*, *Nyctereutes procyonoides*, *Vulpes lagopus*, *Alopex lagopus*, *Cuon alpinus*, *Canis lupus*, including the maned wolf *Chrysocyon brachyurus* (triangles). Data for other canid species were obtained from Duque-Correa et al. (2021), McGrosky et al. (2016), Brudnicki et al. (2011), and Kulawik et al. (2009). SI, small intestine

The gastrointestinal anatomy (Figs. 1, S1–S12) is generally similar to that of other canids. The species has a unilocular (single-chambered), c-shaped stomach. For adults, the stomach had an empty weight of 188 g (Table 1), and greater and lesser curvature lengths of 47 and 17 cm, respectively (Table 2). The small intestine was simple, and the longest out of all intestinal sections. Average length for adults was 323.7 ± 16.1 cm; there was a small cecum without an appendix, and the colon was unsacculated with a uniform diameter, not visibly larger than that of the small intestine (Table 3, Fig. 1). The ratio of small intestine to large intestine length in adult animals was 4.2:1. In the two juveniles, all measures were of lower magnitudes (Tables 1, 2, 3). Compared to published data, the length of intestinal sections of maned wolves was similar to those of other canids, suggesting that relative intestinal length is conserved within the clade (Fig. 2).

**Table 2:** Dimensions (in cm; mean ± SD) of different components of the stomach of maned wolves (*Chrysocyon brachyurus*)

	Juveniles^a^	Adults		
	(*n* = 2)	Females (*n* = 6)	Males (*n* = 4)	Average (*n* = 10)
Fundus diameter	8.5/12.3	12.9 ± 2.4	10.4 ± 2.9	11.9 ± 2.8
Body diameter	11.9/13.1	14.7 ± 3.8	13.0 ± 4.7	14.0 ± 4.0
Pylorus diameter	3.2/3.2	5.6 ± 0.7	5.0 ± 1.5	5.4 ± 1.1
Lesser curvature	15/15	18.0 ± 2.3	15.8 ± 1.4	17.1 ± 2.2
Greater curvature	38/34	51.1 ± 11.4	40.8 ± 11.7	47.0 ± 12.1

^a^For the two juveniles, values of each animal are displayed

**Table 3:** Dimensions (length and diameter in cm; mean ± SD) of different components of the intestinal tract of maned wolves (*Chrysocyon brachyurus*)

	Juveniles^a^	Adults		
	(*n* = 2)	Females (*n* = 6)	Males (*n* = 4)	Average (*n* = 10)
Length				
Small intestine	275.0/286.0	322.1 ± 19.1	326.0 ± 12.5	323.7 ± 16.0
Caecum	5.5/7.3	8.1 ± 1.7	7.8 ± 0.6	8.0 ± 1.3
Colon	67.0/47.0	69.2 ± 6.2	67.0 ± 10.7	68.3 ± 7.8
Large intestine	72.5/54.3	77.3 ± 6.8	74.8 ± 11.0	76.3 ± 8.2
Total intestine	347.5/340.3	399.5 ± 21.1	400.8 ± 23.0	400.1 ± 20.6
Diameter				
Small intestine	1.4/2.8	2.6 ± 0.4	2.9 ± 0.3	2.7 ± 0.4
Caecum	3.4/3.3	3.3 ± 1.1	3.8 ± 1.1	3.5 ± 1.0
Colon	2.3/2.4	2.8 ± 0.3	3.2 ± 0.3	3.0 ± 0.4
Large intestine	2.9/2.9	3.1 ± 0.6	3.5 ± 0.7	3.2 ± 0.6
Total intestine	2.4/2.8	2.9 ± 0.5	3.3 ± 0.5	3.0 ± 0.5

^a^For the two juveniles, values of each animal are displayed. Total intestine diameter is the average measurement from all intestinal sections

**Fig. 3:**
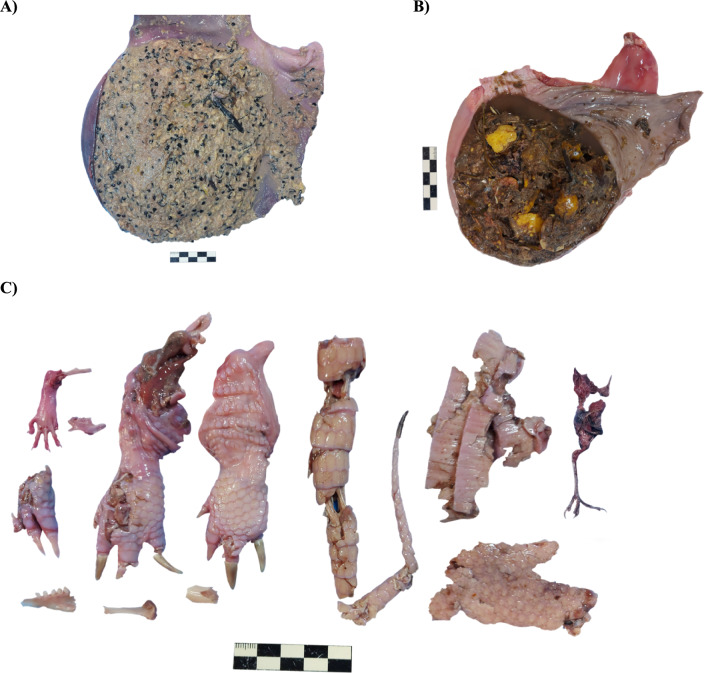
Gastric content of maned wolves (*Chrysocyon brachyurus*). **A** *Solanum lycocarpum* seeds in the stomach of an adult male. **B** fruit remains in the stomach of a juvenile male. **C** partially digested vertebrate remains, rodent foot and jaw (top left), several body parts of an armadillo (*Dasypus* sp.), and a bird foot (top right) found in the stomach of two adult females. Note that section C is composed of the stomach contents of several individuals; see supplementary material. Scale = 5 cm

**Fig. 4:**
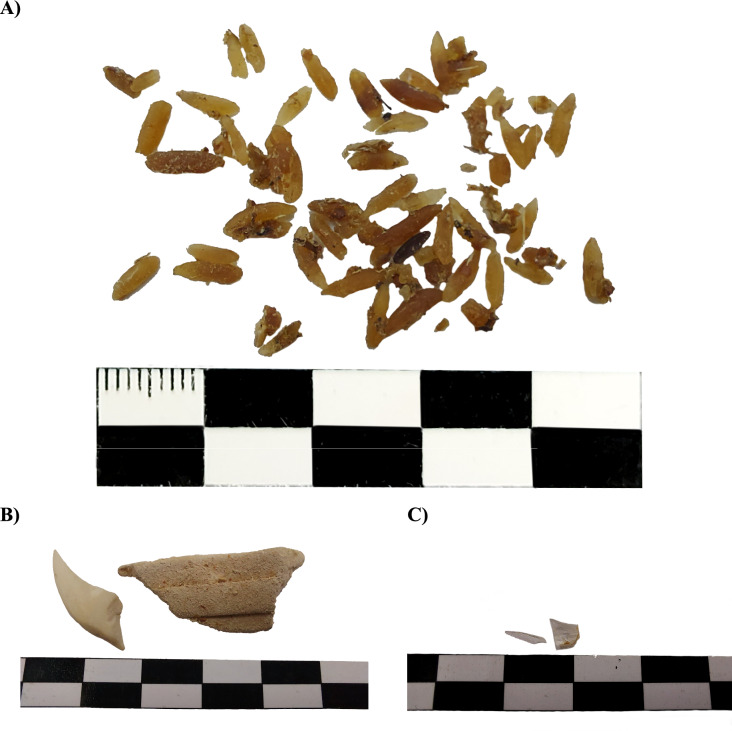
Anthropogenic content retrieved from the stomach of maned wolves (*Chrysocyon brachyurus*). **A** cooked rice. **B** ceramic fragment. **C** glass fragment. Scale = 5 cm

Several vertebrate species were found as prey items and presented as partially digested bone fragments, hair, and other body parts (Figs. 3c, S13–S15, S18, S21–S22). Vertebrates were found in all stomachs (100% FO), representing on average 42.5% of the total dietary mass (Table 4). Among vertebrates, small rodents, small birds, and Xenarthra remains (armadillo osteoderms) were the most frequent items (Figs. 3c, S13–S14, S18, S21). Plant fiber and seeds were often present in the stomach and large intestine (Figs. 3ab, S14b, S16–S17, S20–S23). Vegetable material was present in 100% of stomachs, accounting for half of the total dietary mass (50.5%) (Table 5). *Solanum lycocarpum* was the most common item, with the fruits’ shells, pulp, and seeds commonly found in the gastrointestinal tract (Figs. 3ab, S14, S16, S17, S20–S23). Leaves and grass were also found (Fig. S22). Invertebrates were found in 63% of analyzed stomachs, accounting for only 2.5% of the total consumed volume (Table 4, Fig. S18). Invertebrate prey belonged to the orders Coleoptera, Isoptera, Arachnida, Orthoptera, Homoptera, and Hymenoptera. Finally, anthropogenic, mineral, and non-identified materials were present in a single stomach each, constituting 3.6, 0.1, and 0.8% of the total dietary mass, respectively (Table 4). Although the anthropogenic material was only found in one individual, several items were retrieved, including food (cooked rice) and non-edible material (glass, ceramic and plastic fragments, and human hair) (Fig. 4).

**Table 4:** Diet items identified in the gastrointestinal tract of eight free-ranging maned wolves (*Chrysocyon brachyurus*), relative weight composition, and frequency of occurrence

Categories	Relative weight composition (%)	Frequency of occurrence *n*
Vegetable material	50.5	8 (100%)
Invertebrates	2.5	5 (62.5%)
Vertebrates	42.5	8 (100%)
Anthropogenic material	3.6	1 (12.5%)
Mineral material	0.1	1 (12.5%)
Non-identified material	0.8	3 (37.5%)

**Table 5:** Nutrient analyses (in % of dry matter, mean ± SD) of the gastrointestinal contents of maned wolves (*Chrysocyon brachyurus*) compared to other carnivore species

	Crude protein	Ether extract (Crude fat)	Total ash	Fiber
Maned wolf (*Chrysocyon brachyurus*), free-ranging^a^				
Stomach	43.2 ± 9.5 (*n* = 6)	11.8 ± 4.8 (*n* = 3)	12.3 ± 4.3 (*n* = 3)	44.2 ± 12.8 (*n* = 3)^h^
Small intestine	40.6 ± 13.8 (*n* = 5)	7.4 ± 2.1 (*n* = 4)	8.5 ± 1.9 (*n* = 3)	21.2 ± 12.0 (*n* = 4)^h^
Large intestine	38.3 ± 16.3 (*n* = 6)	6.1 ± 2.3 (*n* = 2)	8.0 ± 2.5 (*n* = 2)	29.8 ± 24.0 (*n* = 3)^h^
Fox (*Vulpes vulpes*), free-ranging^b^				
Stomach	43.3	20.3	13.8	22.6^i^
Raccoon dog (*Nyctereutes procyonoides*), free-ranging^c^				
Stomach	41.4	22.4	12.5	27.9^i^
Wolf (*Canis lupus*), free-ranging^d^				
Theoretical diet	67.2	24.9	6.4	1.5^i^
Domestic cat (*Felis silvestris*), free-ranging^e^				
Theoretical diet	62.7	22.8	11.8	2.7^i^
Maned wolf (*Chrysocyon brachyurus*), zoo^f^				
Prey-based	43–44	16–20	6	2.4–2.6^i^
Extrudate-based	29–30	10–21	10	3.3–3.7^i^
Domestic dog (*Canis familiaris*), recommendations^g^				
Maintenance	10	5.5	–	–
Domestic cat (*Felis silvestris*), recommendations^g^				
Maintenance	20	9	–	–

^a^Present study

^b^Kowalska et al. (2015)

^c^Kowalska et al. (2014)

^d^Bosch et al. (2015)

^e^Plantinga et al. (2011)

^f^Barboza et al. (1994)

^g^NRC (2006)

^h^Neutral detergent fiber (NDF) including residual ash; therefore, the addition of all nutrients to more than 100% is possible

^i^crude fibre

The nutrient analyses of the gastrointestinal contents indicated concentrations of crude protein and ether extract of 43 and 12% of the dry matter in the stomach, and 36 and 4.9% in the large intestine, respectively (Table 5). The ash content was relatively low (12.3–8%) (Table 5). Neutral detergent fiber accounted for 44% of the stomach contents (Table S4).

## Discussion

This study reports quantitative anatomical features of the gastrointestinal tract of maned wolves. We compared the length of intestinal sections with those of other canids from the literature and recorded the dietary and nutrient composition of free-ranging individuals. An evident limitation of the present study is the sample size, determined by the fortuitous collection of carcasses.

Across mammalian taxa, differences in intestinal length can be explained by diet. Species that consume higher proportions of plant matter have longer intestines, especially large intestines (Duque-Correa et al. 2021). However, within the Carnivora, differences in intestinal length are hardly related to diet (McGrosky et al. 2016). When visually comparing the intestinal lengths of several canid species, no distinct differences are detected (Fig. 2). Notably, the species in Fig. 2 included strict faunivores (90–100% animal matter) as well as omnivores (40–60% animal matter) (Wilman et al. 2014). Within mammalian taxa, intestinal length is a phylogenetically conserved feature (Duque-Correa et al. 2021). In particular, the size of the caecum, a typical site of fermentation of difficult-to-digest material such as plant fiber, does not correlate with the proportion of vegetable matter in the diet (McGrosky et al. 2016).

Most dietary analyses of maned wolves’ diet are based on feces, a method most suitable for free-ranging individuals since it is not invasive and fecal material is relatively easy to access (Dietz 1984; Motta‐Junior et al. 1996;; Aragona and Setz 2001). Another alternative for diet assessment is the analysis of stomach contents, a more invasive method since it requires deceased individuals; however, it allows for a more accurate evaluation of consumed items compared to fecal analyses, given that the items can be better identified since they have been exposed to much less digestive action, and easily-digestible foods may not be present in feces (Balestrieri et al. 2011; Pineda-Munoz and Alroy 2014). Even with the methodological differences between the dietary analyses reported here and what has been previously reported for the species, there is good agreement in diet composition. Furthermore, using carcasses of roadkill animals can provide important information about the health status and conditions of the specimens, in addition to being a non-invasive research method (Vieira 2020). Carcasses offer opportunities to explore diet using stable isotope analysis on hair, claws, soft tissues, teeth, and bones to reconstruct diet through an individual's life (Focken 2001). Studies that address the nutritional needs of organisms that make up an ecosystem are tools that help define the functions and expand the knowledge of other biological processes of these animals (Pineda-Munoz and Alroy 2014). Understanding and accessing accurate information about the feeding behavior of different species can provide data for ecomorphology studies and food-web modeling (Pineda-Munoz and Alroy 2014).

The diet of maned wolves is known to be highly seasonal, given the difference in abundance of dietary items across the year (Dietz 1984; Aragona and Setz 2001; Silva and Talamoni 2003). Yet, wolf`s fruit (*S. lycocarpum*) is reported as the most important item in the diet, and it is generally the most consumed plant (Dietz1984; Motta‐Junior et al. 1996; Motta-Junior and Martins 2002; Santos et al. 2003; De Almeida Jácomo et al. 2004). Vegetable material was the second most frequent category in our study (80% FO, 54.5% biomass, Table 4), and the wolf´s fruit was the most abundant and common item.

Among animal matter, mammals are the most consumed; rodents and armadillos are often found in the scats, while insects are seldom consumed (Dietz 1984; Silva and Talamoni 2003; De Almeida Jácomo et al. 2004). Our results support those findings. Vertebrates were found in all analyzed stomachs (100%), while invertebrates were found in only 63% of full stomachs (Table 4). The relative importance of vertebrates and invertebrates in terms of biomass was 42.4 and 2.5%, respectively (Table 4). It has been reported that most vertebrates consumed by maned wolves are of medium and small size (Dietz 1984; Motta‐Junior et al. 1996; Aragona and Setz 2001); in other words, maned wolves do not hunt large prey such as ungulates. We found several small vertebrates in the analyzed stomachs (i.e., rodents, armadillos, and birds), as well as insects. In our limited sample, we did not find evidence of reptile consumption.

The presence of anthropogenic material (cooked rice, glass, ceramic, and plastic fragments, Fig. 4) in one of the stomachs indicates opportunistic feeding behavior. Aragona and Setz (2001) already observed this behavior in the Ibitipoca State Park. This result confirms the presence of the species in areas inhabited by humans, creating potential for conflict in terms of nutritional impacts due to change of diet, the consumption of potentially harmful items like glass or ceramic, increased risk of disturbance by humans and exposure to diseases, if maned wolves come in contact with domestic dogs (Consorte-McCrea 2013; Aximoff et al. 2020).

To our knowledge, no nutrient data on free-ranging maned wolves have been published so far, and the only other nutrient composition analysis was done on zoo-kept individuals (Barboza et al. 1994). Protein content was similar between wild and zoo diets; however, fat was higher, and fiber lower in the zoo diet. It should be noted that contrary to most dietary analyses, the fiber results presented here contain ash; hence, comparisons with other studies should be interpreted considering this limitation (Tables 5 and S4). When comparing the data from the free-ranging maned wolves of the present study to the zoo diets, it appears that it is particularly the fiber that has not been considered so far. Rather than only focusing on protein, the relevance of fiber for zoo animals should be further elucidated. The nutrient composition of the stomach contents of free-ranging maned wolves was of similar magnitudes as those reported for foxes (*Vulpes vulpes*) and raccoon dogs (*Nyctereutes procyonoides*), all canid species considered omnivores (Table 5). By contrast, the nutrient composition of two faunivorous carnivorans (canid: grey wolf *Canis lupus*; felid: feral *Felis silvestris*) are characterized by distinctively higher protein and lower fiber levels (Table 5). Regarding feeding recommendations for zoo-kept maned wolves, Barboza et al. (1994) suggested that crude protein levels of ‘prey-based diets’ were too high for maned wolves and that, rather, low-protein diets based on extrudates should be used instead. Similarly, Allen (1995) recommended that maned wolves in zoos should receive diets with a maximum crude protein content of 28% dry matter. Notably, maintenance protein requirements for domestic dogs are low (Table 5), yet such low levels are hardly fed to dogs. In other words, domestic dogs consistently consume diets higher in protein than required. Similarly, when comparing the experimentally derived maintenance protein recommendations for domestic dogs and cats to the protein levels of the natural diets of wolves and feral cats (Table 5), it is evident that diets of a distinctively higher protein level than deemed necessary are typical for carnivorans. Possibly, conventional zoo diets that have been described as high in protein have perceived negative effects not because of their protein but because of their low fiber content.

## Conclusion

Our study describes the morphology of the gastrointestinal tract of free-ranging maned wolves and the analysis of gastrointestinal contents. The general anatomy and the dimensions of the maned wolf`s GIT resembled other canids. As expected, dietary analyses confirm the omnivorous nature of the species, consuming both vegetable and animal material. The wolf`s fruit (*S. lycocarpum*) and small mammals are the most important dietary sources, respectively. The presence of anthropogenic material in one individual's stomach indicates opportunistic behavior and habitat overlapping with human settlements.

Dietary studies of wild species can be considered one of the primary factors for improving conservation and management actions. Concerning ‘omnivorous’ Carnivora, especially the fiber level of natural diets should receive further attention. Furthermore, the information obtained through these studies can reveal data related to ecological interactions between species, as well as evolutionary, physiological, and behavioral aspects, and allow for a greater understanding of the trophic structure of the ecosystem.

## Supplementary Information

Below is the link to the electronic supplementary material.

Supplementary file1 (PDF 2395 KB)

## Data Availability

All data generated or analyzed during this study are included in this published article and its supplementary information files.
