# Supplementary material for: Digestive anatomy and diet of free-ranging maned wolf (Chrysocyon brachyurus)
Source: Mamm Biol. 2025 Apr 24;105(4):479–88. doi: 10.1007/s42991-025-00493-z (PMC12287139; doi:10.1007/s42991-025-00493-z)
Supplement: Supplementary file 1 — Supplementary file1 (PDF 2395 KB) [file 42991_2025_493_MOESM1_ESM.pdf]

Supplementary Material

Digestive anatomy and diet of free-ranging maned wolf (*Chrysocyon brachyurus*)

Bruno Costa Silva[1] • Luan Alexander de Oliveira[2] • Marcus Clauss[3] • Claudia Guimarães Costa[4] • Leandro de Oliveira Marques Alexandre[4] • María J. Duque-Correa[3*]

[1]Departamento de Medicina Veterinária - Pontifícia Universidade Católica de Minas Gerais, Belo Horizonte, Brazil

[2]Programa de Pós-graduação em Biodiversidade e Meio Ambiente - Pontifícia Universidade Católica de Minas Gerais, Av. Dom José Gaspar, 500, Belo Horizonte, Brazil

[3]Clinic for Zoo Animals, Exotic Pets and Wildlife, Vetsuisse Faculty, University of Zurich, Winterthurerstrasse 260, 8057 Zurich, Switzerland

[4]Coleção de Mastozoologia, Museu de Ciências Naturais da Pontifícia Universidade Católica de Minas, Belo Horizonte, Brazil

BCS, brunobiopsy@yahoo.com.br, 0000-0002-0543-6095
LAO, luanalexanderdeoliveira@gmail.com, 0009-0000-9634-4120
MC, mclauss@vetclinics.uzh.ch, 0000-0003-3841-6207
CGC, cacau@pucminas.br, 0000-0002-9348-8708
LOM, leandromarques@pucminas.br, 0000-0003-3265-0414
MJD-C, mariajose.duquecorrea@uzh.ch, 0000-0001-8431-2228

✉María J. Duque-Correa

mariajose.duquecorrea@uzh.ch

SUPPLEMENTARY INFORMATION

Table S1: Basic information of the free-ranging maned wolves (*Chrysocyon brachyurus*) specimens included in this study, showing which examinations were carried out.

Animal	ID	Sex	Collection date	Coordinates	Anatomical description	Stomach content	Bromatological essay
Female 1	LOM 820	Female	28.12.2020	20° 06' 34"S 44° 19' 04"W	X	X	X
Female 2	LOM 821	Female			X	X	X
Female 3	LOM 822	Female			X		
Female 4	LOM 841	Female	02.09.2020	22º 17' 16,4"S 45º 53' 50,2"W	X	X	X
Female 5	LOM 856	Female	27.09.2021	20° 09' 51"S 44° 21' 59"W	X		X
Female 6	LOM 945	Female	04.09.2022	20° 13' 32"S 44° 23' 42"W	X		
Female 7	LOM 254	Female	18.09.2012	20° 10' 11"S 43° 57' 57"W		X	
Female 8	LOM 498	Female	01.12.2015	20° 09' 30"S 44° 02' 49"W		X	
Female 9	LOM 634	Female	29.12.2019	20º 11' 45.8"S 44º 25' 05.7"W		X	
Male 1	LOM 814	Male		19° 12' 46.7"S 42° 18' 22.9"W	X	X	X
Male 2	LOM 819	Male	17.12.2020	20° 43' 46"S 44° 46' 21"W	X		X
Male 3	BC 01	Male			X		
Male 4	LOM 936	Male			X	X	
Juvenile 1	LOM 875	Male			X		X

Juvenile 2	LOM 874	Male	23.03.2021	20° 10' 11"S 43° 57' 57"W	X		X

Table S2: Mass (in g; mean ± SD) of the contents of the different components of the gastrointestinal tract (GIT) of the maned wolf (*Chrysocyon brachyurus*).

	Juveniles* (n=2)	Adults Females (n=6)	Males (n=4)	Average (n=10)
Body mass (kg)	6.4/13.3	20.7 ± 4.7	19.2 ± 4.6	20.1 ± 4.5
Stomach	0/310	301.5 ± 414.2	256.0 ± 459.3	283.3 ± 407.8
Small intestine	65/420	165.5 ± 79.5	122.5 ± 53.4	148.3 ± 53.4
Caecum	2/0	6.3 ± 8.8	4.8 ± 6.2	5.7 ± 7.5
Colon	40/40	39.5 ± 46.9	99.7 ± 101.1	63.6 ± 74.8
Large intestine	42/40	45.8 ± 52.7	104.5 ± 106.9	69.3 ± 79.2
Total intestine	107/460	211.3 ± 119.1	227.0 ± 105.9	217.6 ± 108.1
Total GIT	107/770	512.8 ± 476.1	483.1 ± 478.3	500.9 ± 468.8

*for the two juveniles, values of each animal are displayed

Table S3: Weight (g) of the stomach contents retrieved divided by feeding categories from the stomachs of free-ranging maned wolves (*Chrysocyon brachyurus*).

Animal	Feeding categories (g) Vegetable material	Invertebrates	Vertebrates	Anthropogenic material	Non-identified material
Female 1	0.12	-	6.97	-	-
Female 2	30.13	0.21	2.67	-	-
Female 4	0.17	-	5.53	-	-
Female 7	38.92	-	0.98	-	-
Female 8	4.04	4.77	6.09	8.40	0.97
Female 9	35.08	0.04	62.72	-	-
Male 2	5.41	0.81	2.20	-	0.50
Male 4	4.70	0.05	12.58	-	0.35

Table S4: Nutrient analyses (in % of dry matter, mean ± SD) of the gastrointestinal contents of free-ranging maned wolves (*Chrysocyon brachyurus*).

Animal	Sample site	Crude protein	Neutral detergent fiber* (NDF)	Ether extract (crude fat)	Total ash
Female 1	Stomach	54.5	51.99	13.14	16.86
Female 1	SI	48.07	12.79	7.84	-
Female 1	LI	41.62	8.09	4.52	9.82
Female 2	Stomach	45.43	27.04	14.64	8.36
Female 2	SI	44.53	15.29	5.46	7.59
Female 2	LI	33.53	25.865	-	-
Female 4	Stomach	49.13	-	-	-
Female 4	LI	64.35	-	-	-
Female 5	SI	55.81	-	-	-
Juvenile 1	SI	34.34	17.75	10.20	10.73
Juvenile 2	Stomach	24.85	40.83	7.52	11.81
Juvenile 2	SI	20.16	38.92	6.28	7.34
Juvenile 2	LI	14.01	55.66	7.75	6.2
Male 1	Stomach	43.67			
Male 1	LI	34.57			
Male 2	Stomach	47.44			
Male 2	LI	41.82			

SI: small intestine
LI: large intestine
*Neutral detergent fiber (NDF), including residual ash; therefore, addition of all nutrients to more than 100% is possible

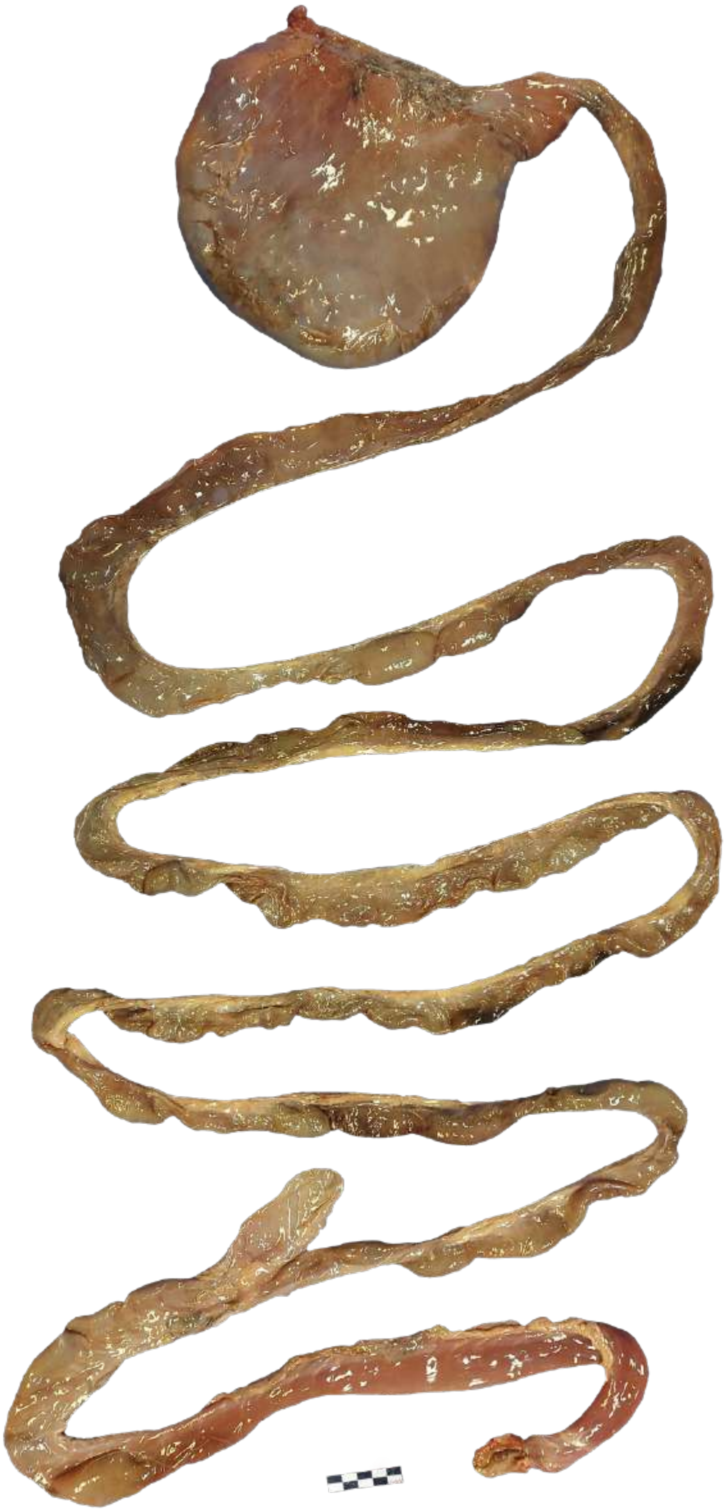

Figure S1: Gastrointestinal anatomy of a free-ranging maned wolf (*Chrysocyon brachyurus*), female 1. Scale = 5 cm.

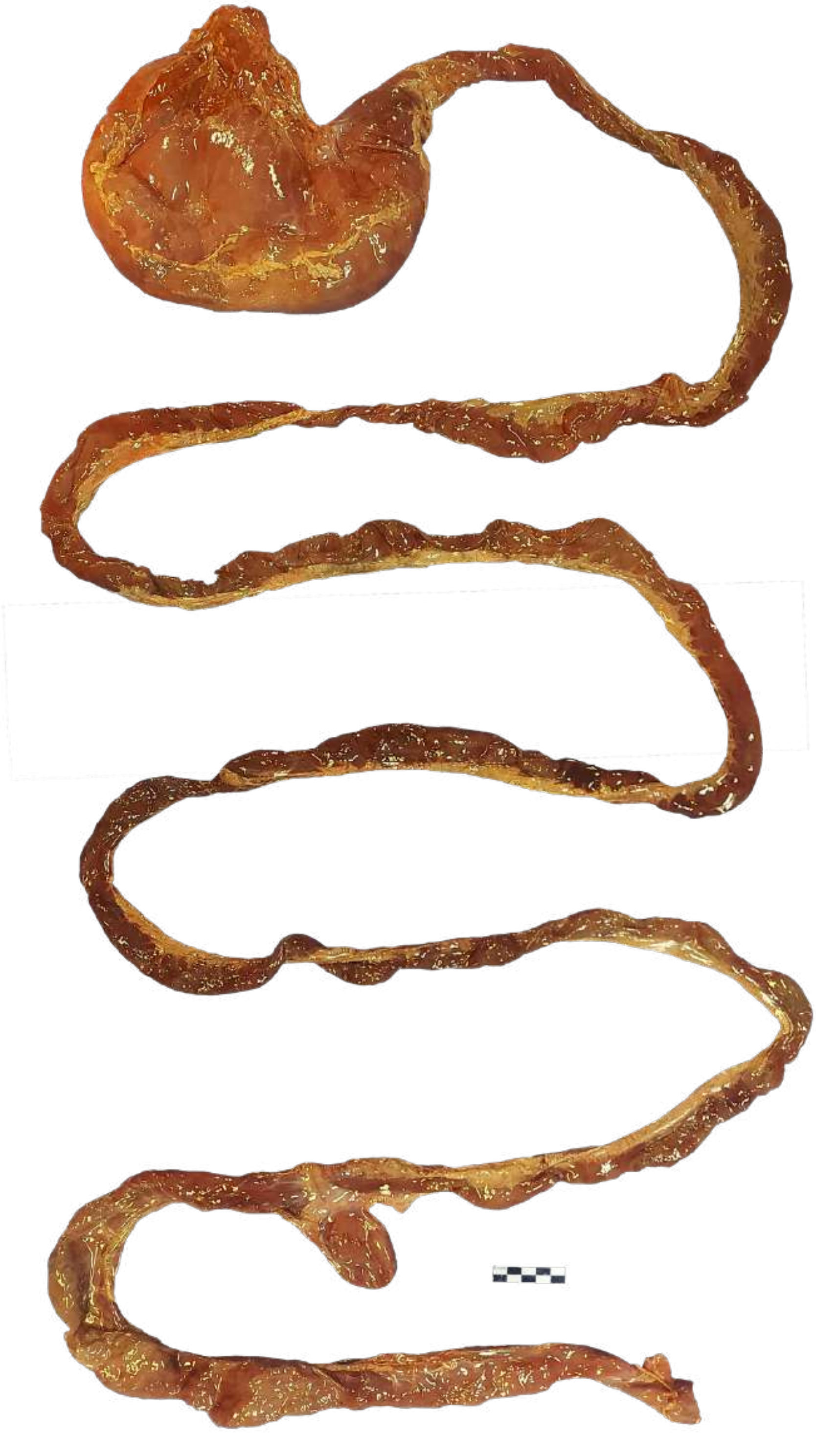

Figure S2: Gastrointestinal anatomy of a free-ranging maned wolf (*Chrysocyon brachyurus*), female 2. Scale = 5 cm.

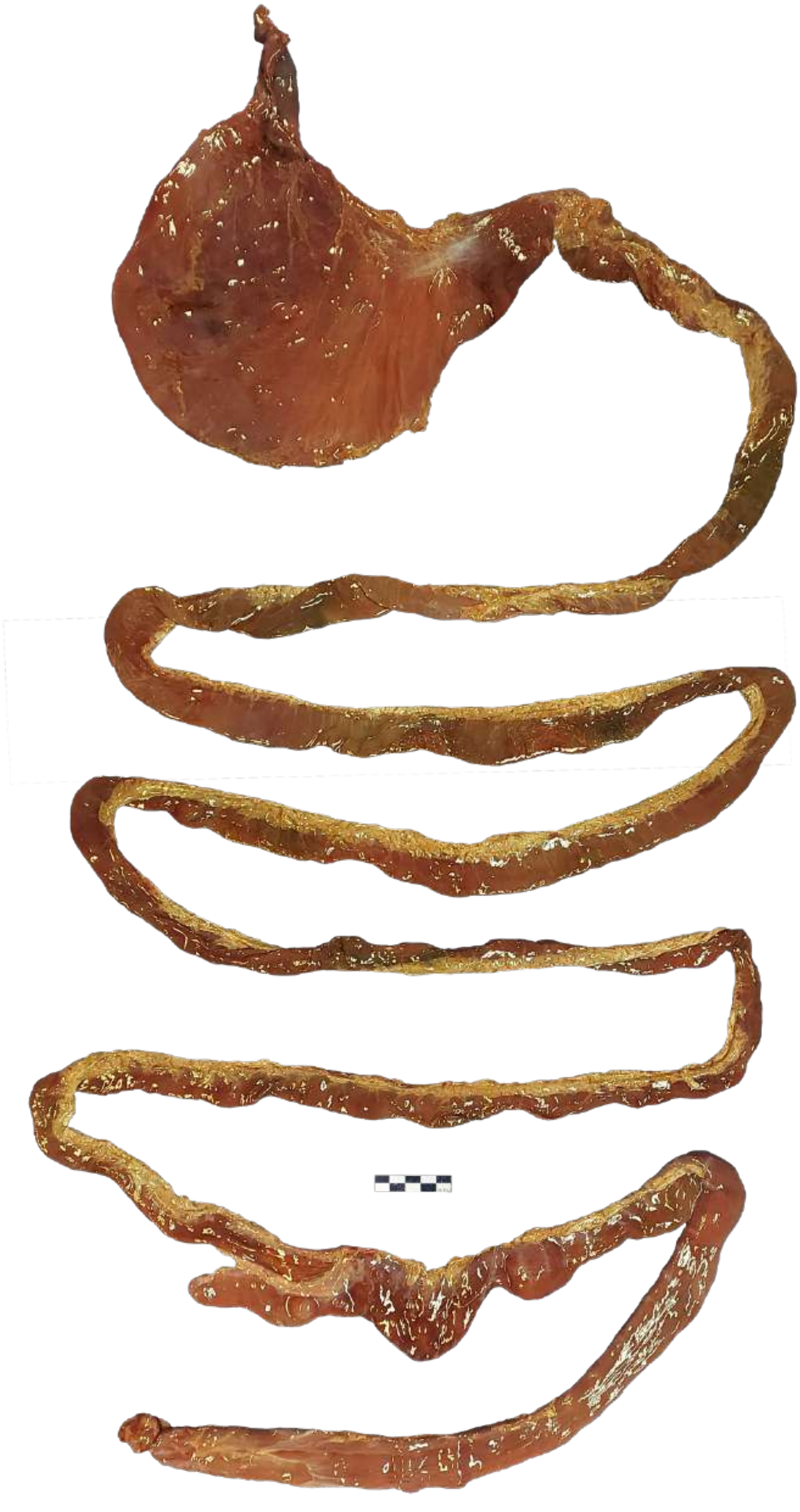

Figure S3: Gastrointestinal anatomy of a free-ranging maned wolf (*Chrysocyon brachyurus*), female 3. Scale = 5 cm.

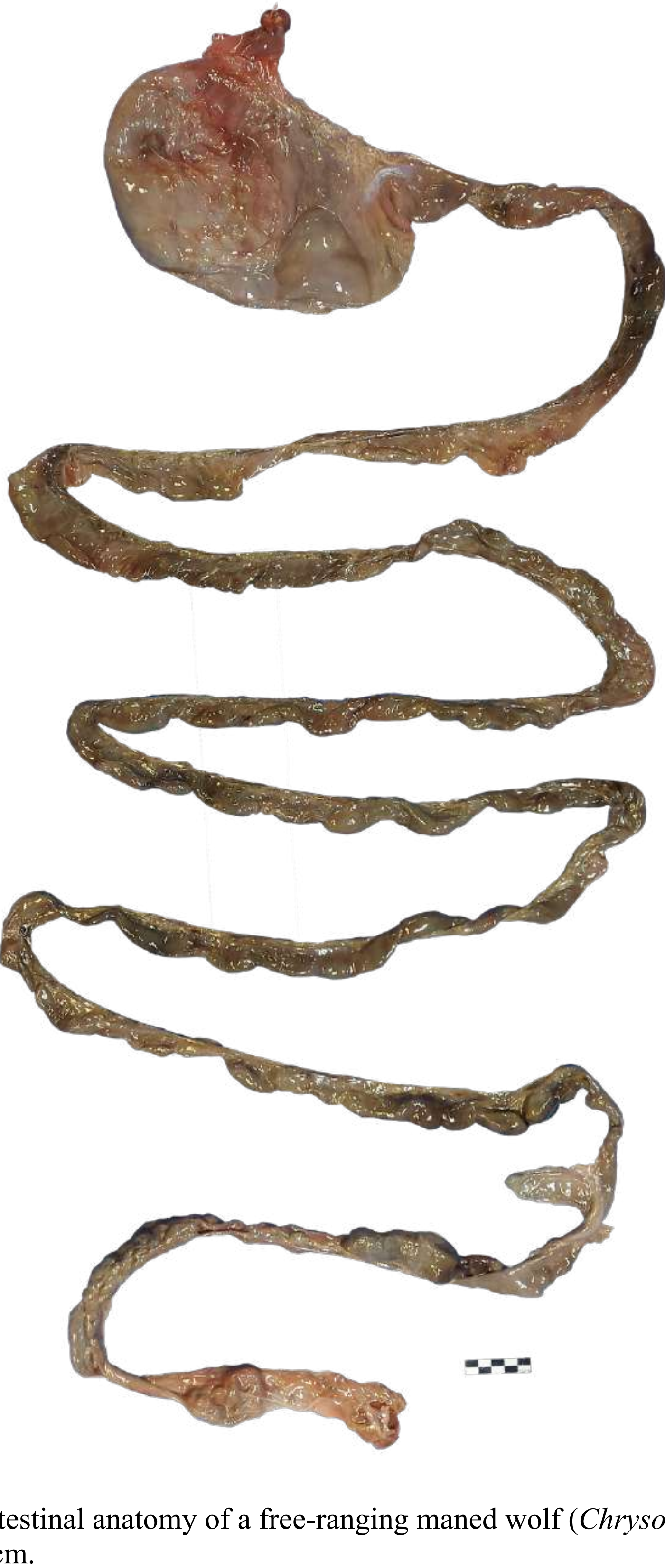

Figure S4: Gastrointestinal anatomy of a free-ranging maned wolf (*Chrysocyon brachyurus*), female 4. Scale = 5 cm.

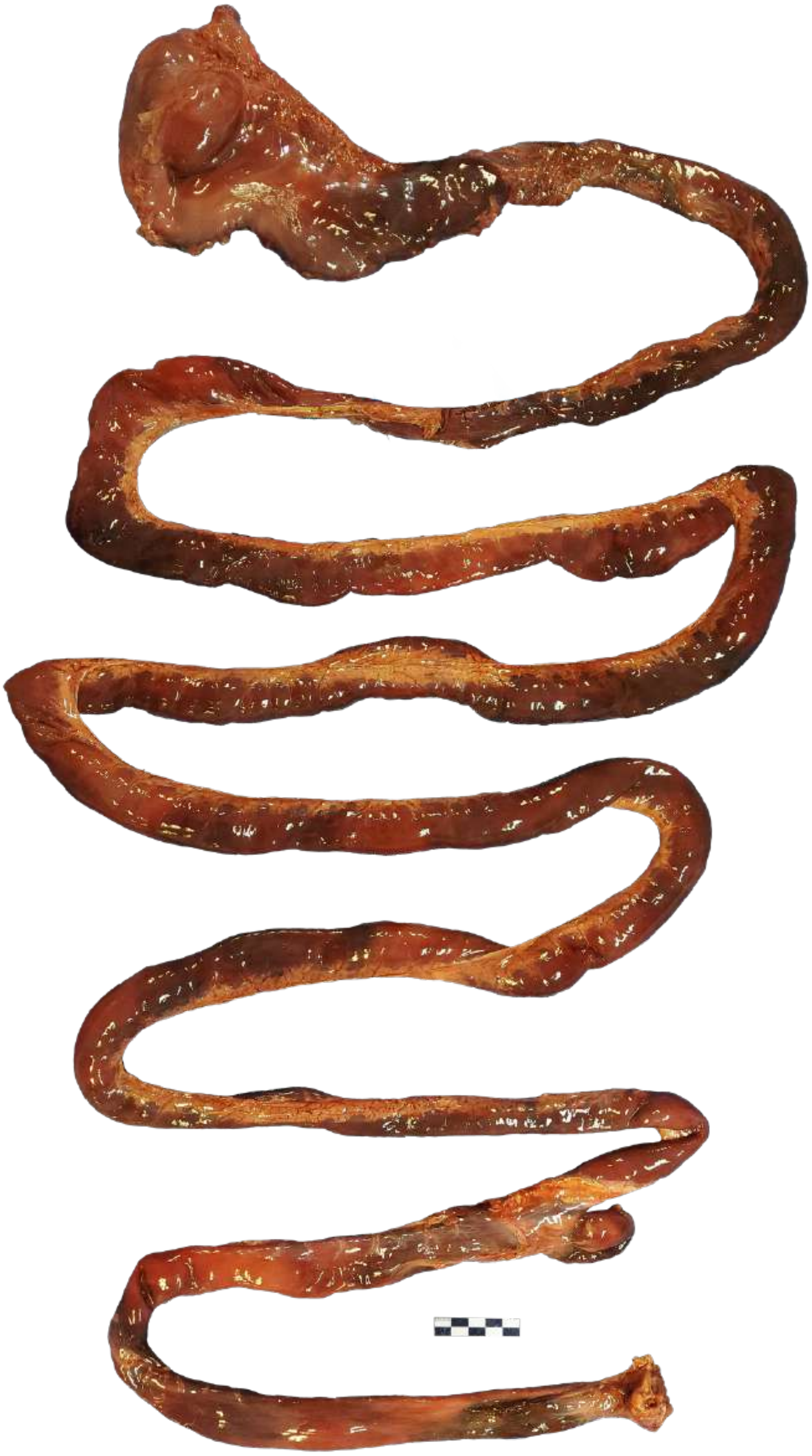

Figure S5: Gastrointestinal anatomy of a free-ranging maned wolf (*Chrysocyon brachyurus*), female 5. Scale = 5 cm.

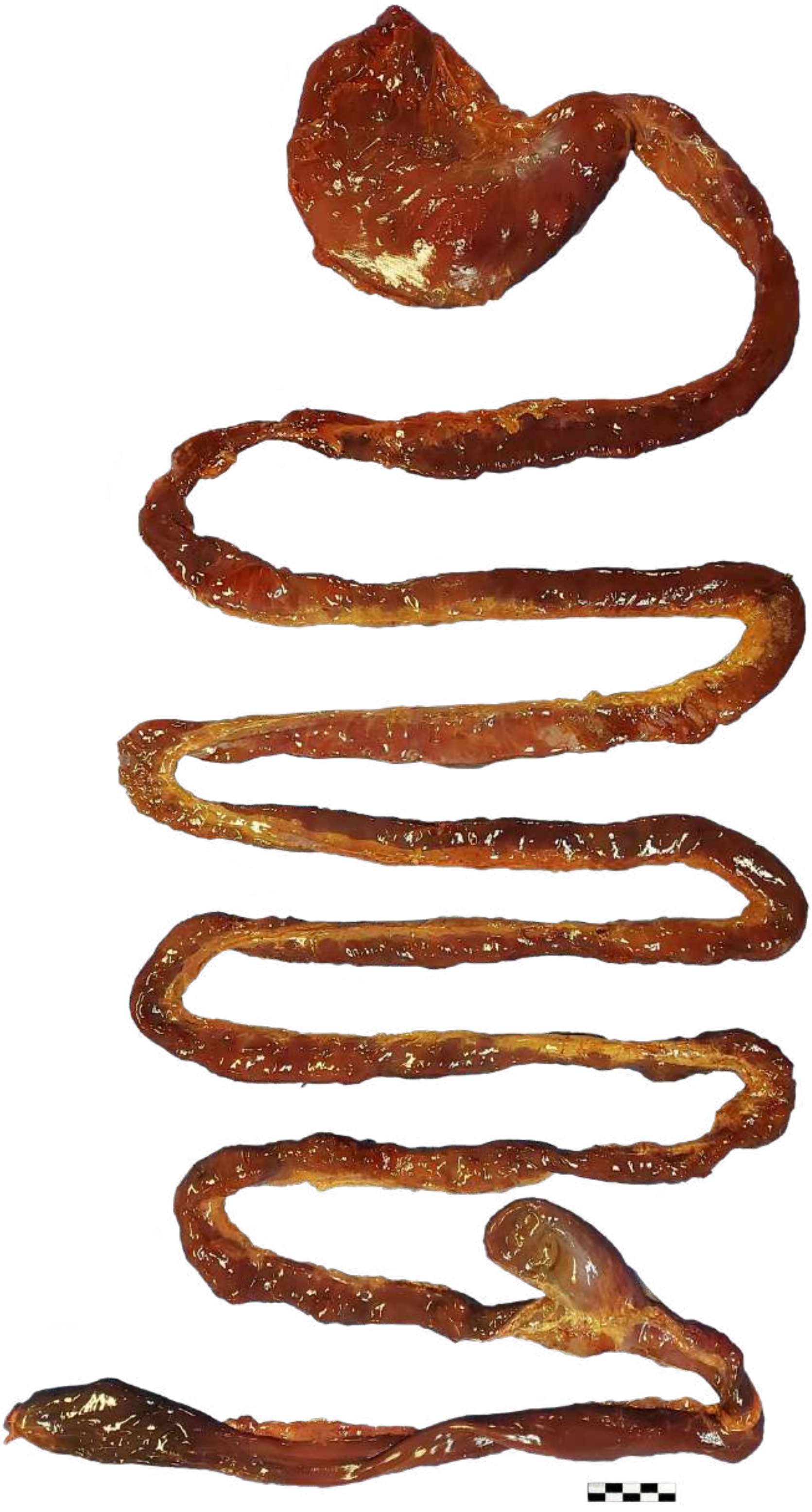

Figure S6: Gastrointestinal anatomy of a free-ranging maned wolf (*Chrysocyon brachyurus*), female 6. Scale = 5 cm

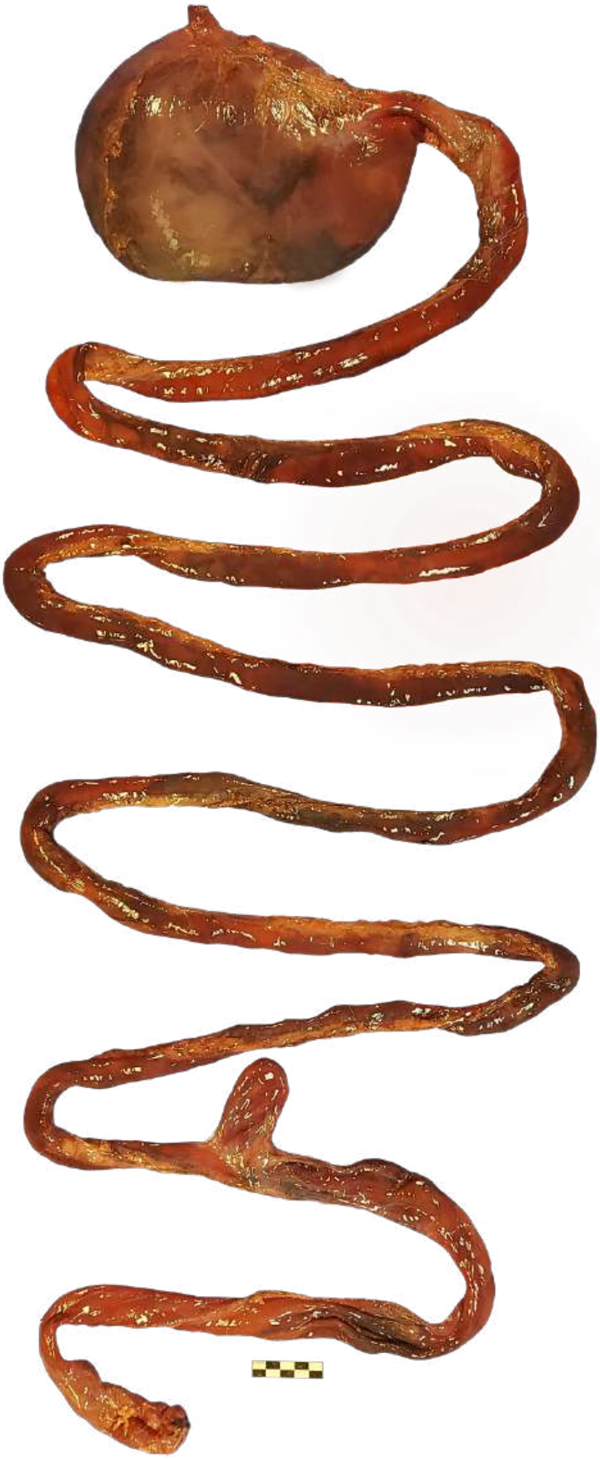

Figure S7: Gastrointestinal anatomy of a free-ranging maned wolf (*Chrysocyon brachyurus*), male 1. Scale = 5 cm.

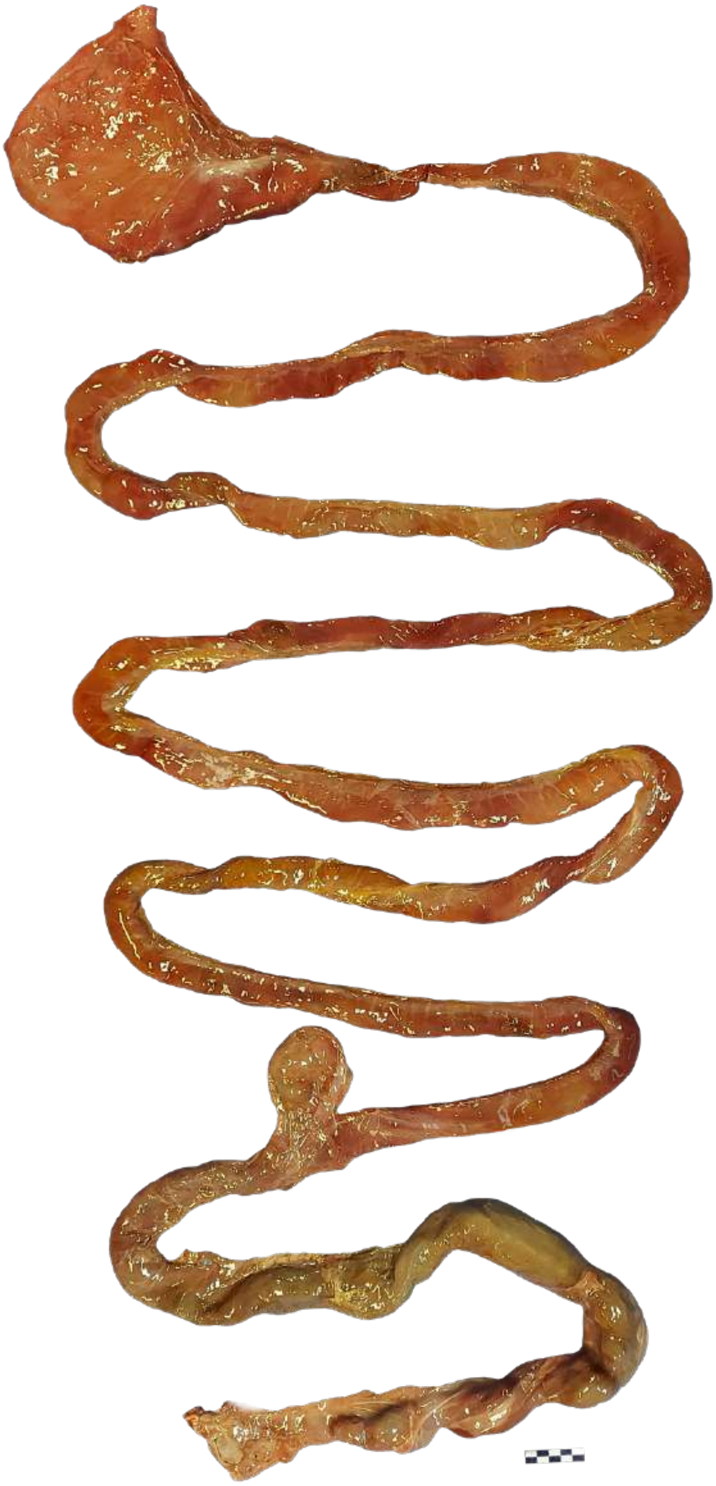

Figure S8: Gastrointestinal anatomy of a free-ranging maned wolf (*Chrysocyon brachyurus*), male 2. Scale = 5 cm.

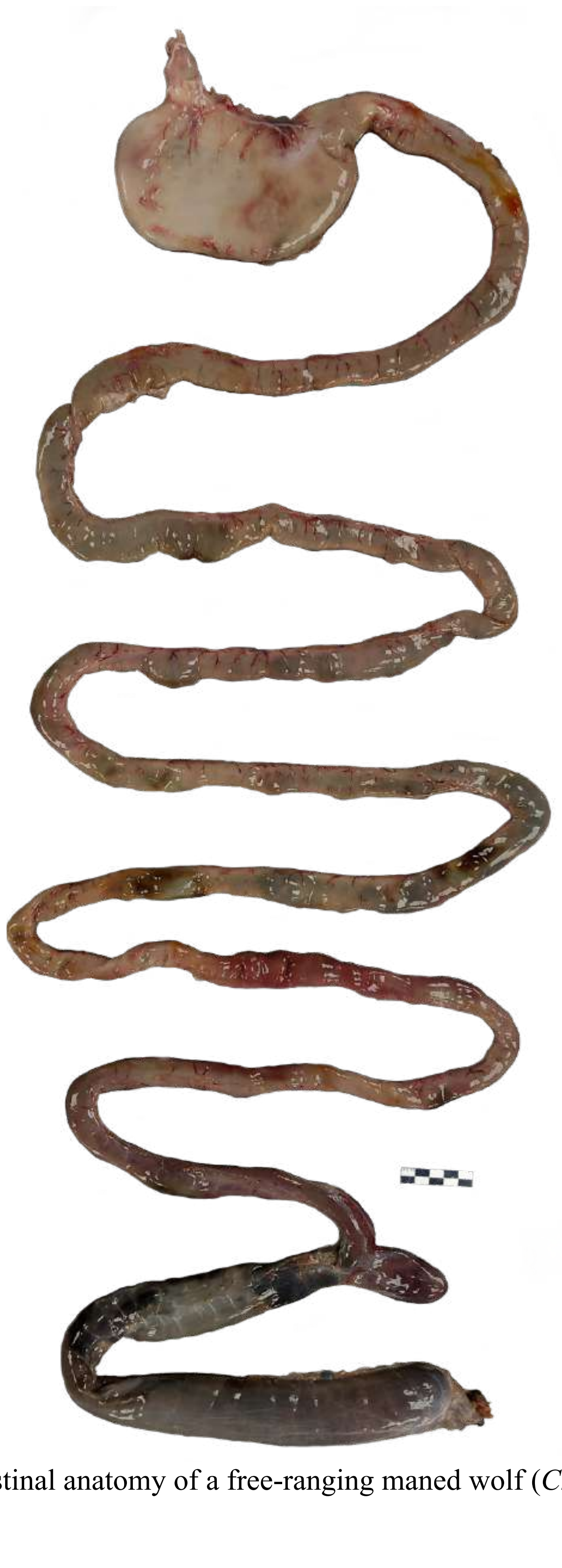

Figure S9: Gastrointestinal anatomy of a free-ranging maned wolf (*Chrysocyon brachyurus*), male 3. Scale = 5 cm.

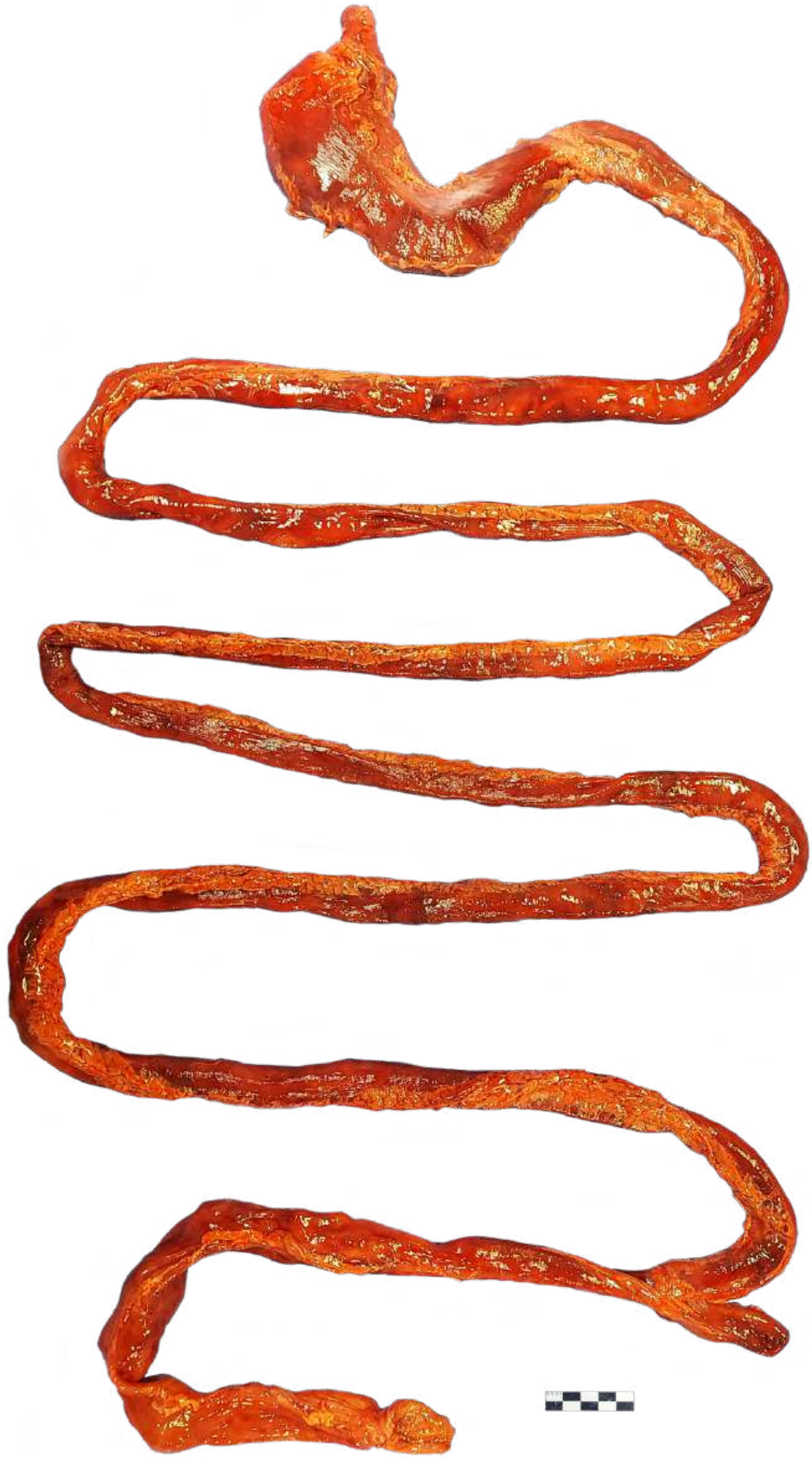

Figure S10: Gastrointestinal anatomy of a free-ranging maned wolf (*Chrysocyon brachyurus*), male 4. Scale = 5 cm.

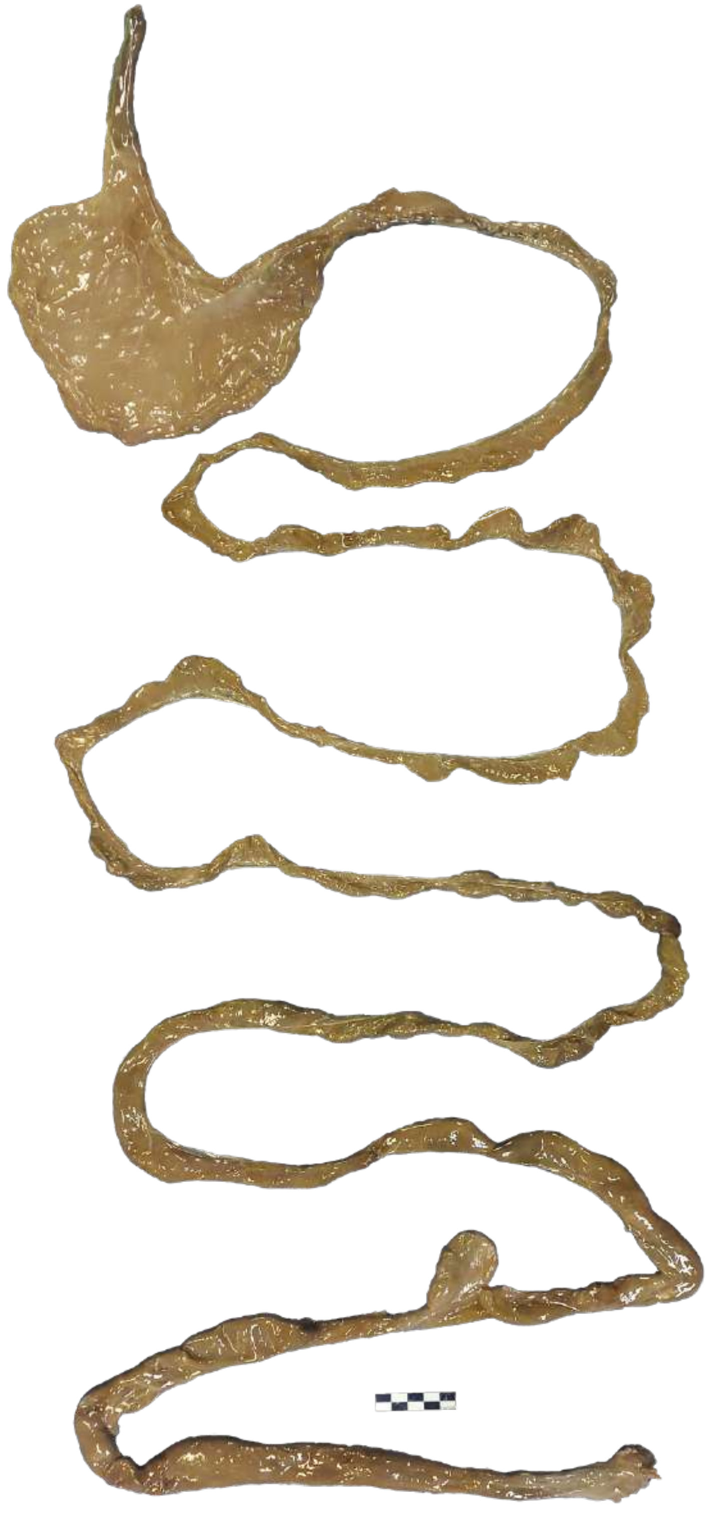

Figure S11: Gastrointestinal anatomy of a free-ranging maned wolf (*Chrysocyon brachyurus*), juvenile 1. Scale = 5 cm.

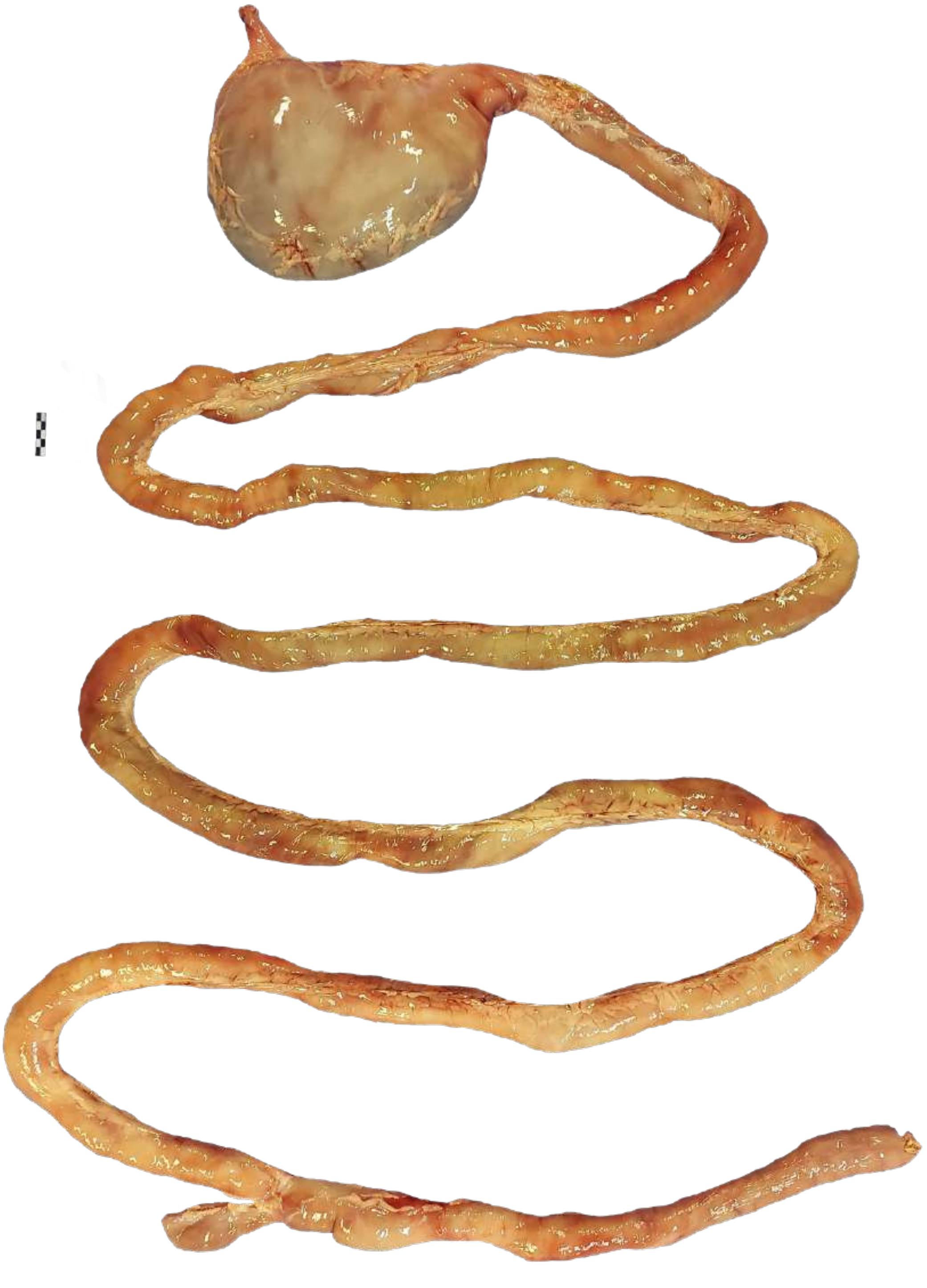

Figure S12: Gastrointestinal anatomy of a free-ranging maned wolf (*Chrysocyon brachyurus*), juvenile 2. Scale = 5 cm.

A)

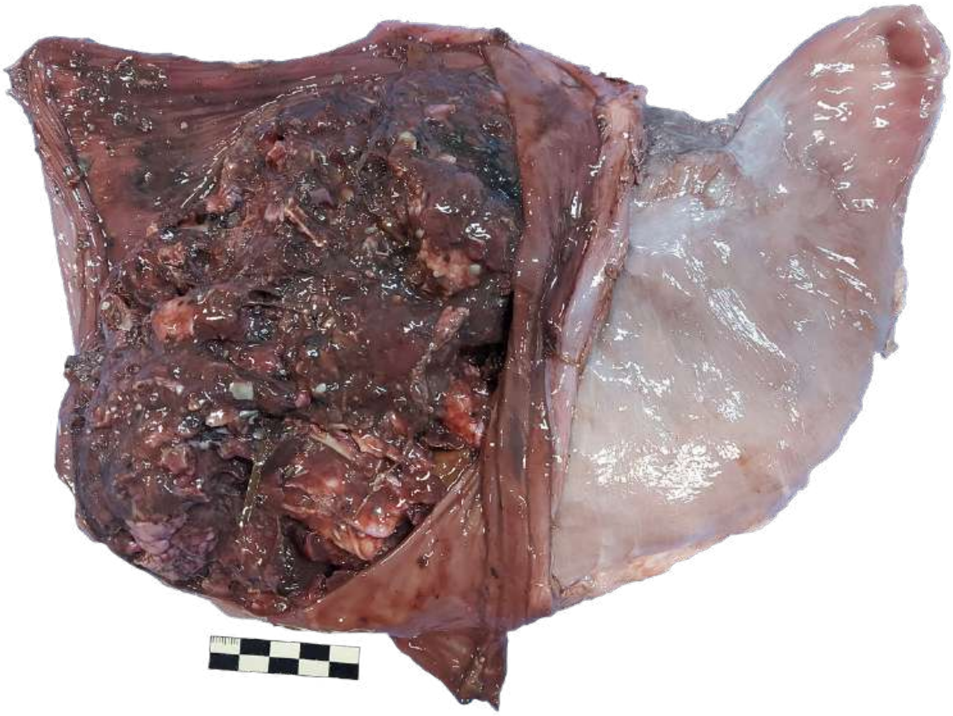

B)

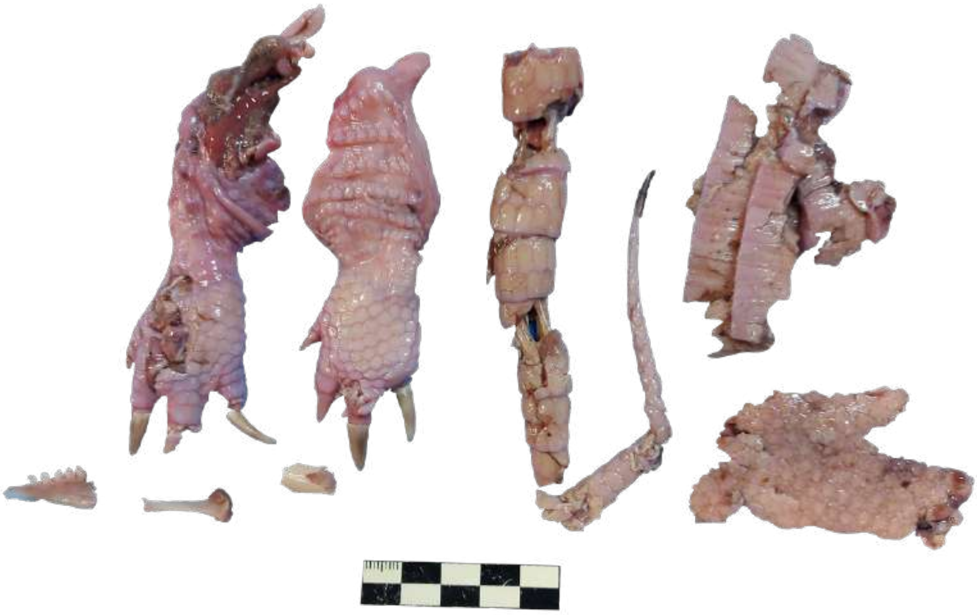

Figure S13: Gastric content of a free-ranging maned wolf (*Chrysocyon brachyurus*), female 1. A) Partially opened stomach with evidence of consumption of vertebrates (visible bone fragments and osteoderms). B) Partially digested vertebrate remains, several body parts of an armadillo (*Dasypus* sp.). Scale= 5 cm.

A)

B)

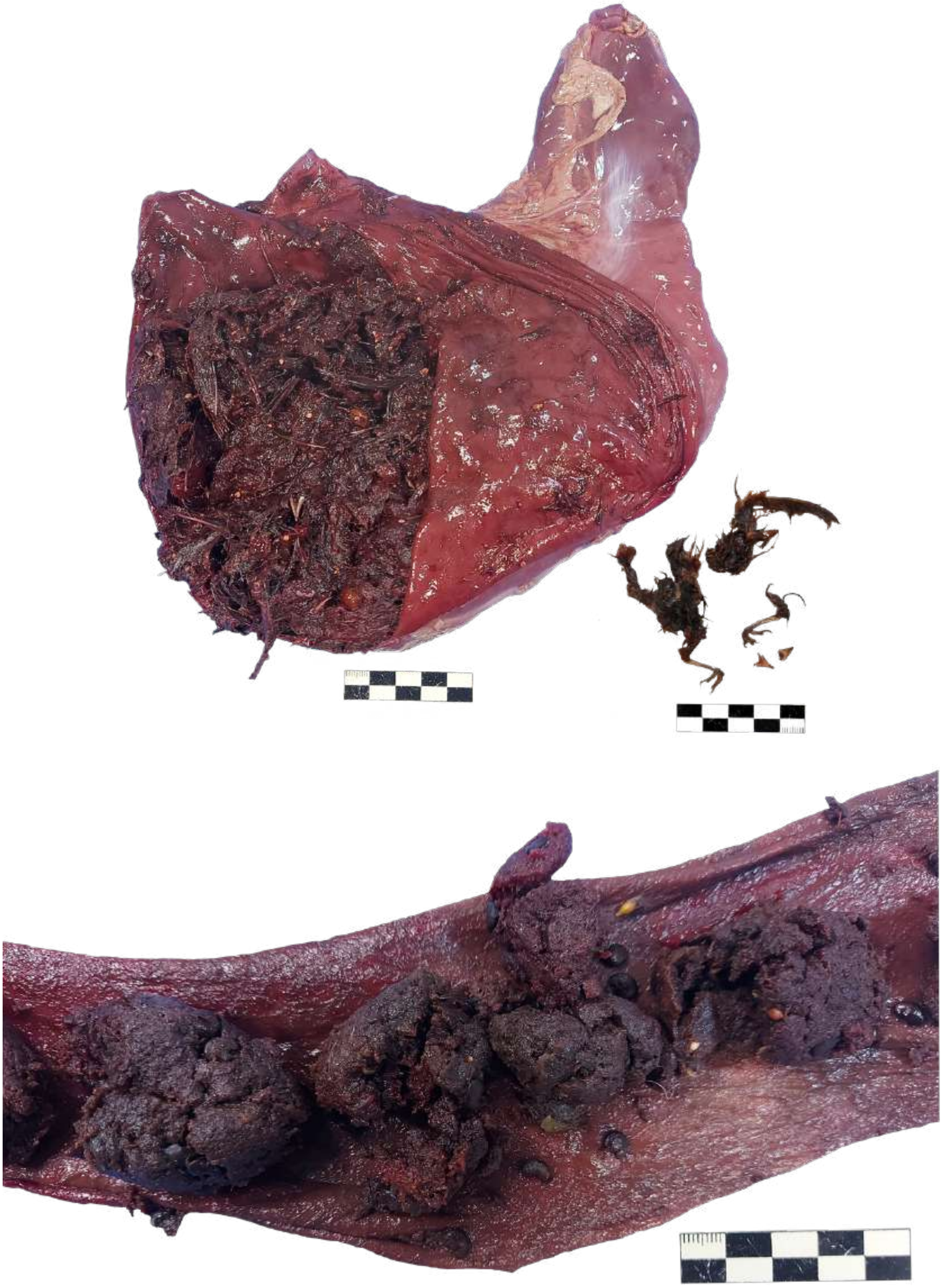

Figure S14: Gastrointestinal content of a free-ranging maned wolf (*Chrysocyon brachyurus*), female 2. A) Partially opened stomach with evidence of consumption of vertebrates (bottom right: retrieved stomach contents, bird’s beak, legs, and feathers). B) Formed feces in the colon with undigested hair and wolf’s fruit seeds (*Solanum lycocarpum*). Scale= 5 cm.

A)

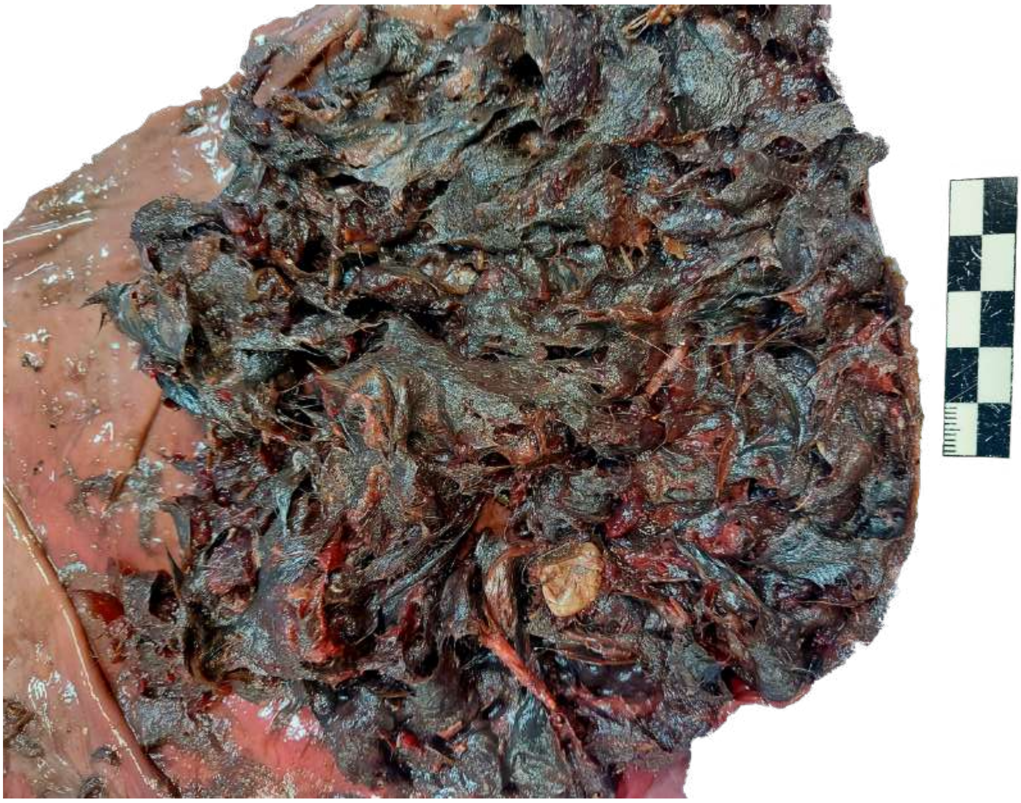

B)

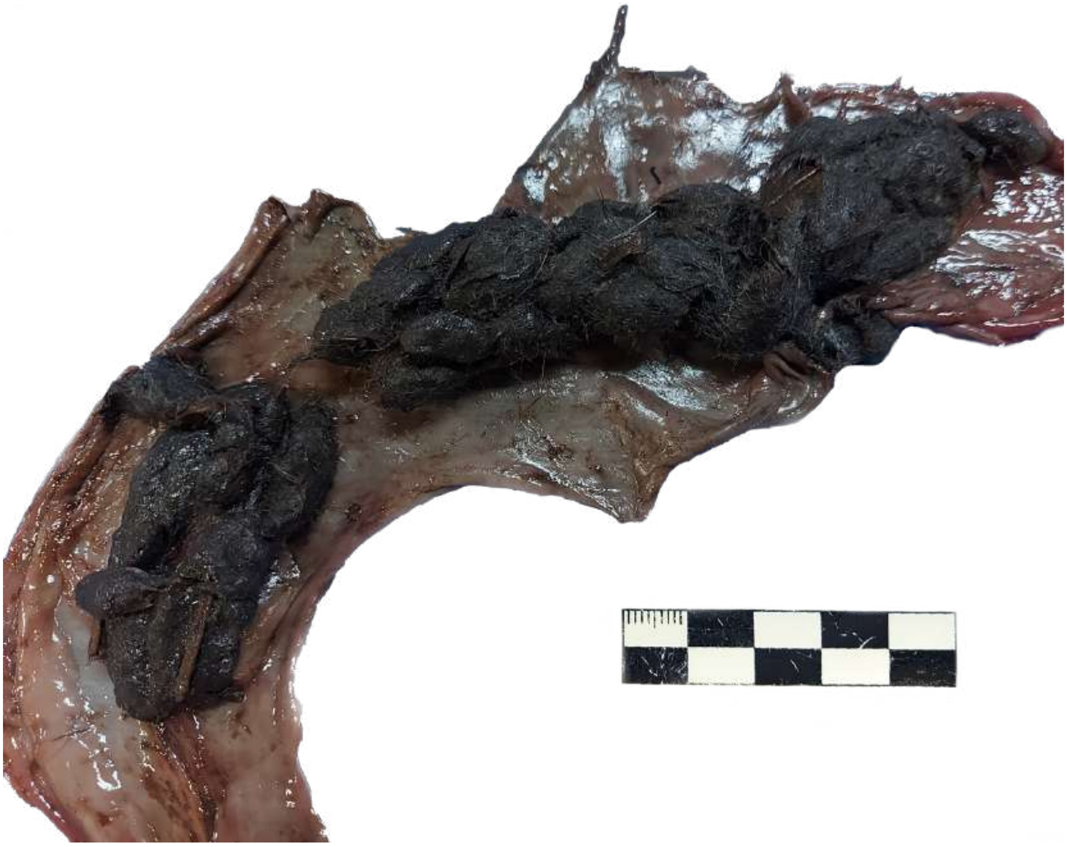

Figure S15: Gastrointestinal content of a free-ranging maned wolf (*Chrysocyon brachyurus*), female 4. A) Partially opened stomach with evidence of consumption of vertebrates (hair). B) Formed feces in the colon with undigested hair and vegetable particles. Scale= 5 cm.

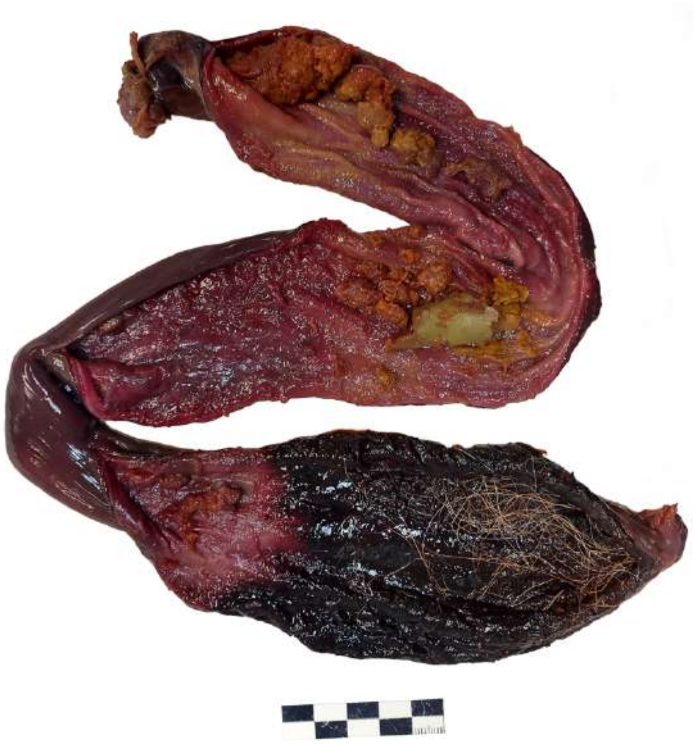

Figure S16: Intestinal content of a free-ranging maned wolf (*Chrysocyon brachyurus*), female 6. Partially opened colon with evidence of wolf's fruit (*Solanum lycocarpum*, undigested shell) and vertebrate consumption (undigested bone fragment) and hair. Scale= 5 cm.

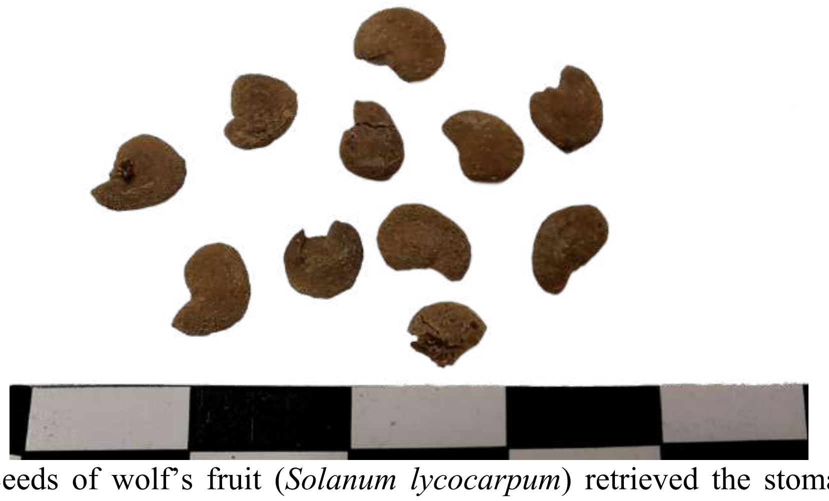

Figure S17: Seeds of wolf's fruit (*Solanum lycocarpum*) retrieved the stomach of a free-ranging maned wolf (*Chrysocyon brachyurus*), female 7. Scale= each rectangle equals one cm.

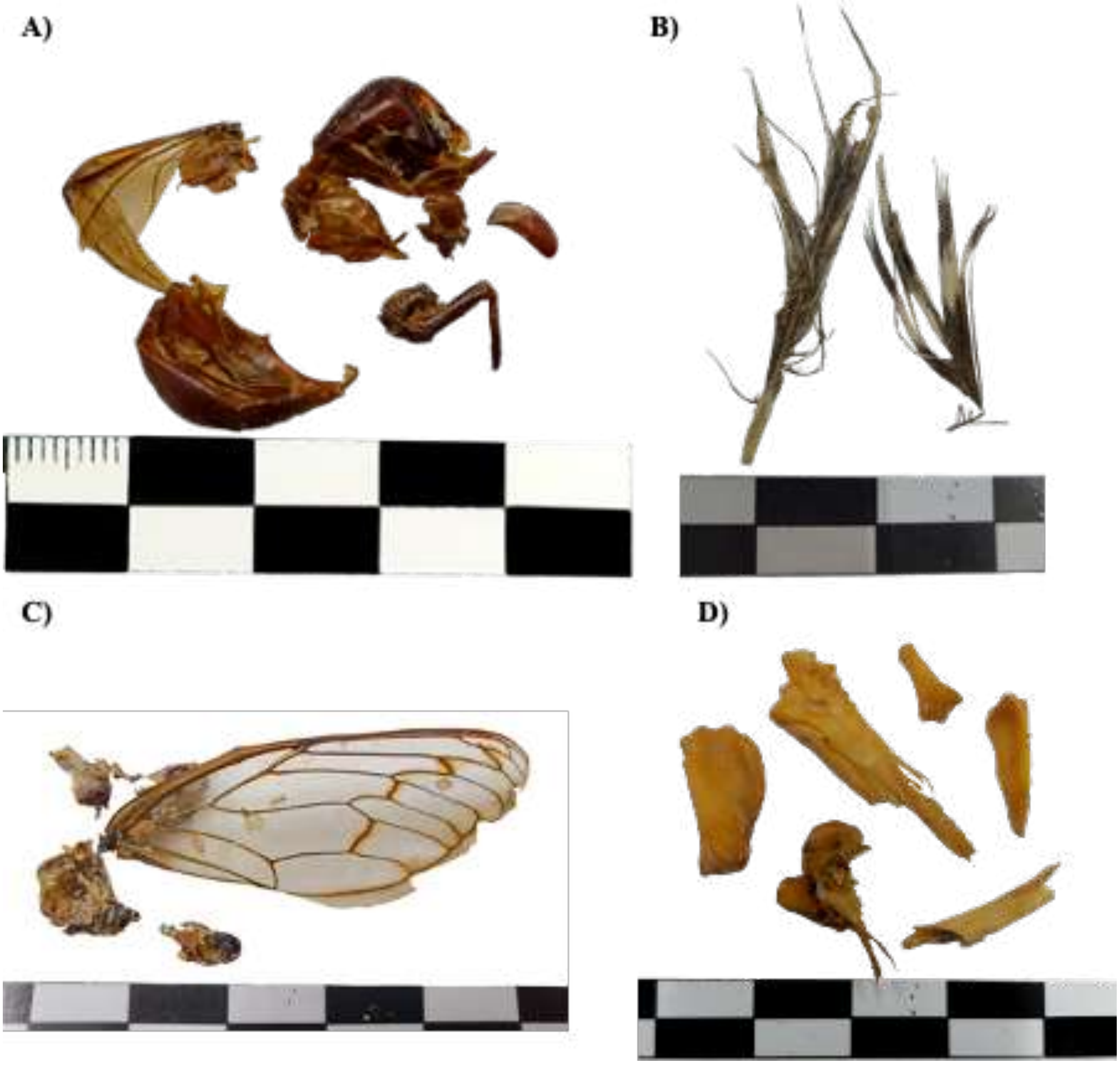

Figure S18: Gastrointestinal content of a free-ranging maned wolf (*Chrysocyon brachyurus*), female 8. Evidence of vertebrate and invertebrate consumption A) Partially digested Coleoptera. B) Feathers. C) Partially digested Hemiptera. D) Bone fragments. Scale= 5 cm.

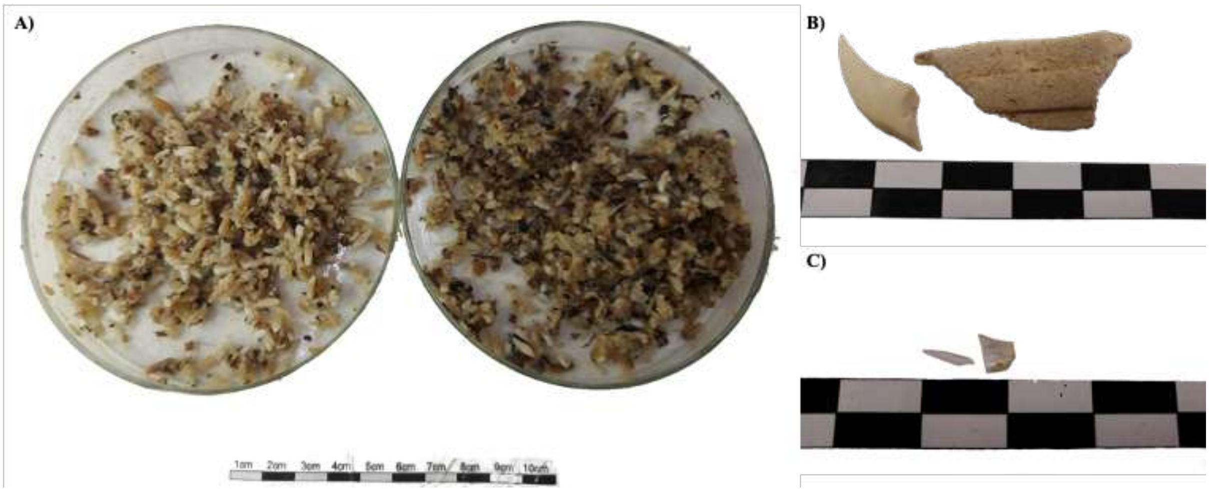

Figure S19: Anthropogenic content retrieved from the stomach of a free-ranging maned wolf (*Chrysocyon brachyurus*), female 8. A) Cooked rice. B) Ceramic fragments. C) Glass fragments. Scale= each rectangle is one centimeter.

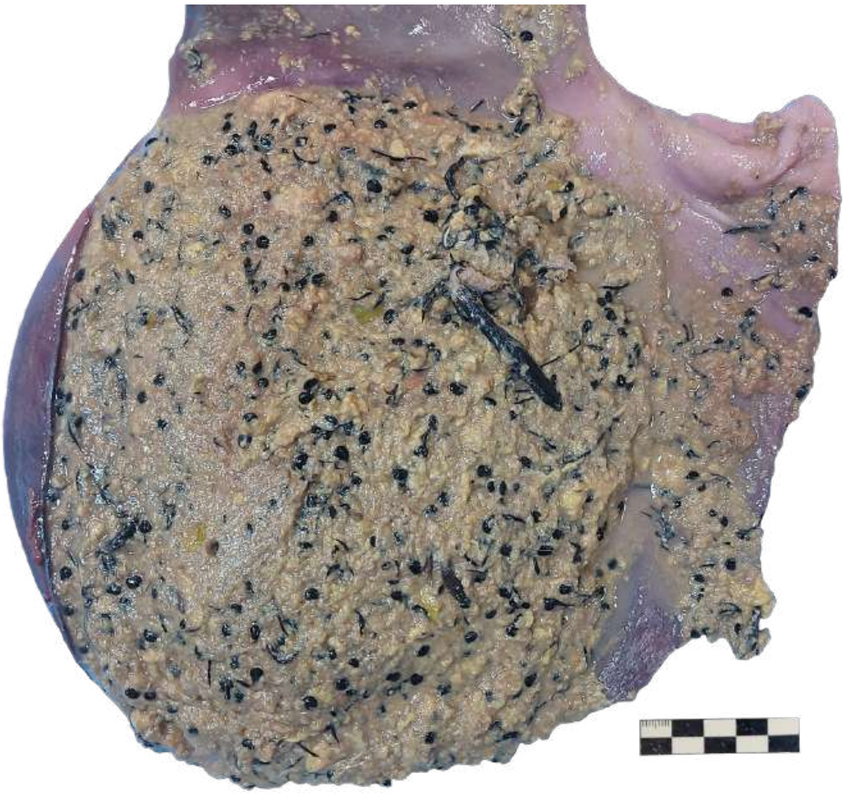

Figure S20: Gastric content of a free-ranging maned wolf (*Chrysocyon brachyurus*), male 1. Evidence of wolf's fruit (*Solanum lycocarpum*, seeds*)* consumption. Scale= 5 cm.

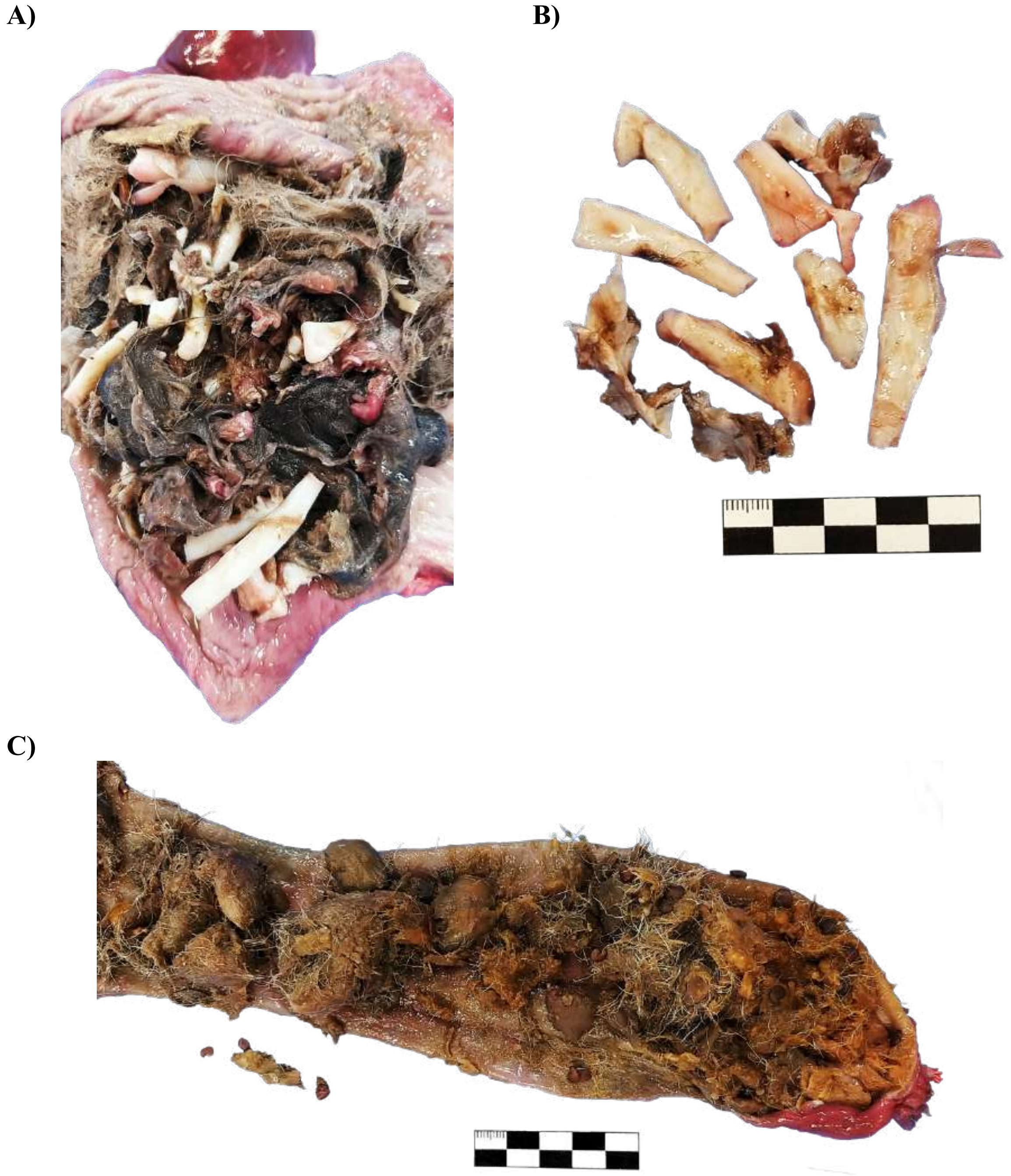

Figure S21: Gastrointestinal content of a free-ranging maned wolf (*Chrysocyon brachyurus*), male 2. A) Partially opened stomach with evidence of rodent consumption (hair and cartilage). B) Bone fragments and cartilage retrieved from the stomach. C) Evidence of wolf's fruit (*Solanum lycocarpum*, seeds) and vertebrate (hair) consumption in the colon. Scale= 5 cm.

A)

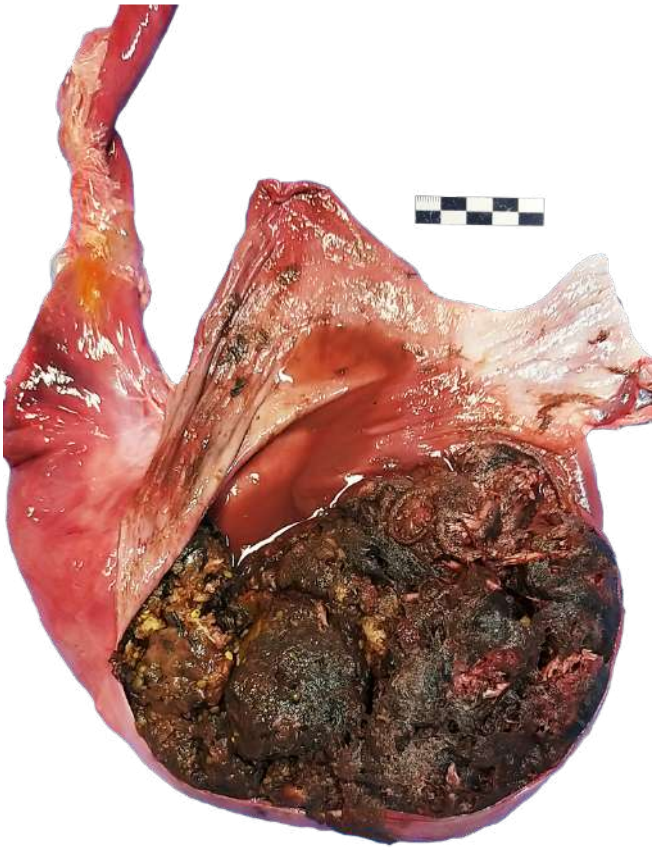

B)

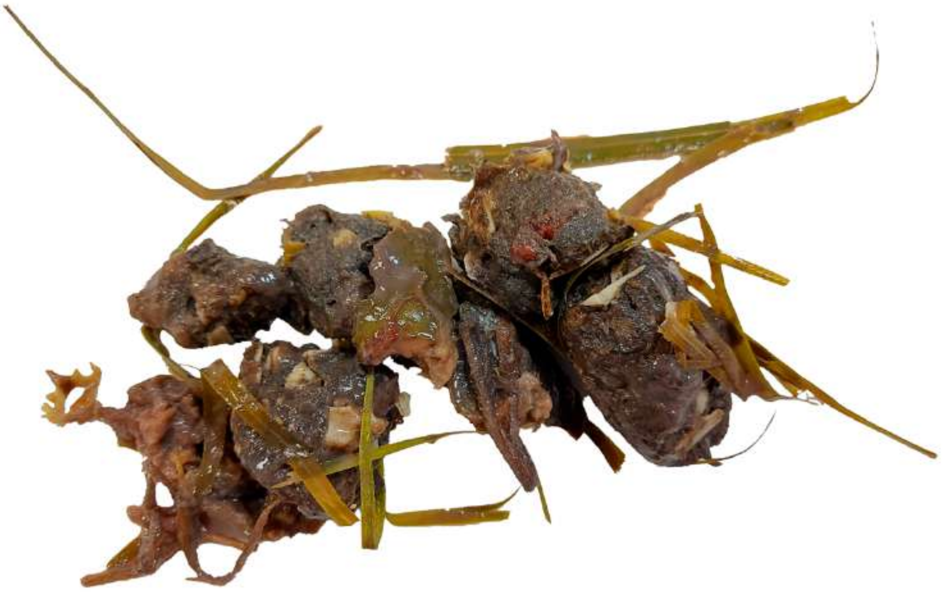

Figure S22: Gastric content of a free-ranging maned wolf (*Chrysocyon brachyurus*), male 4. A) Partially opened stomach with evidence of rodent consumption (hair and partially digested body parts) and of wolf's fruit (*Solanum lycocarpum*, seeds). B) Feces with evidence of vertebrates (bone fragments), grass (capim), and wolf's fruit (*S. lycocarpum,* shell and seeds present*)* consumption. Scale= 5 cm.

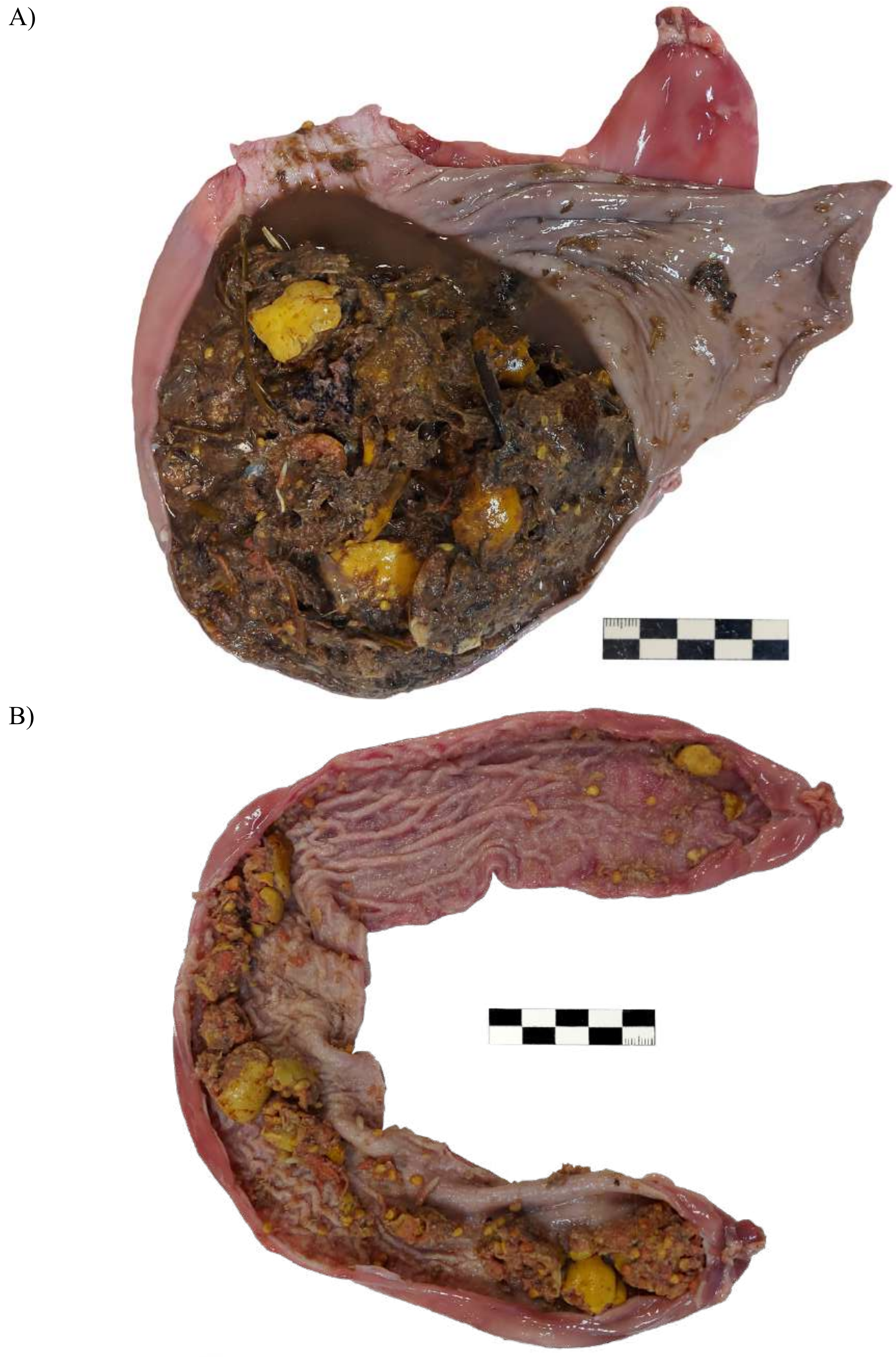

Figure S23: Gastrointestinal content of a free-ranging maned wolf (*Chrysocyon brachyurus*), juvenile 2. A) Partially opened stomach with evidence of wolf's fruit (*Solanum lycocarpum,* seeds, and shell fragments) and grass consumption. B) Opened colon with evidence of consumption of wolf's fruit (*S. lycocarpum,* seeds, and shell fragments). Scale = 5 cm.
